# New Synthetic Thrombin Inhibitors: Molecular Design and Experimental Verification

**DOI:** 10.1371/journal.pone.0019969

**Published:** 2011-05-16

**Authors:** Elena I. Sinauridze, Alexey N. Romanov, Irina V. Gribkova, Olga A. Kondakova, Stepan S. Surov, Aleksander S. Gorbatenko, Andrey A. Butylin, Mikhail Yu. Monakov, Alexey A. Bogolyubov, Yuryi V. Kuznetsov, Vladimir B. Sulimov, Fazoyl I. Ataullakhanov

**Affiliations:** 1 Laboratory of Physical Biochemistry, National Research Center for Hematology, Russian Academy of Medical Sciences, Moscow, Russia; 2 Research Computer Center, Moscow State University, Moscow, Russia; 3 Laboratory of Biophysics and Physiology of Cell, Center for Theoretical Problems of Physicochemical Pharmacology, Russian Academy of Sciences, Moscow, Russia; 4 Physics Department, Moscow State University, Moscow, Russia; 5 Institute of Organic Chemistry, Russian Academy of Sciences, Moscow, Russia; 6 Dimonta, Ltd., Moscow, Russia; Aston University, United Kingdom

## Abstract

**Background:**

The development of new anticoagulants is an important goal for the improvement of thromboses treatments.

**Objectives:**

The design, synthesis and experimental testing of new safe and effective small molecule direct thrombin inhibitors for intravenous administration.

**Methods:**

Computer-aided molecular design of new thrombin inhibitors was performed using our original docking program SOL, which is based on the genetic algorithm of global energy minimization in the framework of a Merck Molecular Force Field. This program takes into account the effects of solvent. The designed molecules with the best scoring functions (calculated binding energies) were synthesized and their thrombin inhibitory activity evaluated experimentally *in vitro* using a chromogenic substrate in a buffer system and using a thrombin generation test in isolated plasma and *in vivo* using the newly developed model of hemodilution-induced hypercoagulation in rats. The acute toxicities of the most promising new thrombin inhibitors were evaluated in mice, and their stabilities in aqueous solutions were measured.

**Results:**

New compounds that are both effective direct thrombin inhibitors (the best K_I_ was <1 nM) and strong anticoagulants in plasma (an IC_50_ in the thrombin generation assay of approximately 100 nM) were discovered. These compounds contain one of the following new residues as the basic fragment: isothiuronium, 4-aminopyridinium, or 2-aminothiazolinium. LD_50_ values for the best new inhibitors ranged from 166.7 to >1111.1 mg/kg. A plasma-substituting solution supplemented with one of the new inhibitors prevented hypercoagulation in the rat model of hemodilution-induced hypercoagulation. Activities of the best new inhibitors in physiological saline (1 µM solutions) were stable after sterilization by autoclaving, and the inhibitors remained stable at long-term storage over more than 1.5 years at room temperature and at 4°C.

**Conclusions:**

The high efficacy, stability and low acute toxicity reveal that the inhibitors that were developed may be promising for potential medical applications.

## Introduction

Hemostasis is one of the most important processes in organisms, and disorders in this system cause deaths under a variety of pathologies. The activation of blood coagulation can be caused by trauma, sepsis, inflammation, obstetric practice and in the course of surgical operations, especially operations using extracorporal blood circulation. Hypercoagulation has also been observed during infusion therapy with large volumes of crystalloid plasma substitutes [1], [2]. Oral contraception and artificial vessels or cardiac valves may be sources of minor but permanent activation of coagulation, eventually exhausting the pool of coagulation inhibitors and giving rise to thrombotic events.

Thrombotic pathologies are a result of an imbalance in the activity of thrombin, a key enzyme of the coagulation cascade, and its natural inhibitors. Overproduction of thrombin may be countered by the administration of drugs that specifically inhibit this enzyme.

This simplified conception allows for the design of new drugs through the development of organic compounds that are inhibitors for the given target-protein. An ideal inhibitor should be highly effective and safe, and it should have stable pharmacokinetics that are only weakly dependent on the patient's age, sex, concomitant diseases, drugs and diet. The binding of a compound with plasma proteins may also interfere with its inhibitory activity. From all these points of view, synthetic inhibitors with a low molecular weight are very promising [3]. Thus, a lot of studies have been directed towards the discovery of effective and safe small molecule anticoagulants that act via direct thrombin inhibition. However, despite considerable attention in this area, only one synthetic direct thrombin inhibitor (DTI), argatroban [4], is currently in use for intravenous administration in medicine. Dabigatran etexilat was approved recently as the first small molecule thrombin inhibitor for peroral introduction [5]. Thus, the development of effective new direct thrombin inhibitors is a very important objective for the improvement of anticoagulant therapy.

This study presents the results of our search for new small molecule thrombin inhibitors for intravenous administration.

New inhibitor design is one of the key phases of the long and expensive process of developing new drugs. The structures of thrombin and many of its complexes with a diverse set of experimental inhibitors have been resolved by X-ray structure analysis, and many of these 3D structures have been submitted to the Protein Data Bank (PDB) [6]. This information, together with modern methods of structure-based drug design, can be used to shorten the discovery and design phases of new drug development concerning by completing a search for new inhibitor structures.

**Figure 1:**
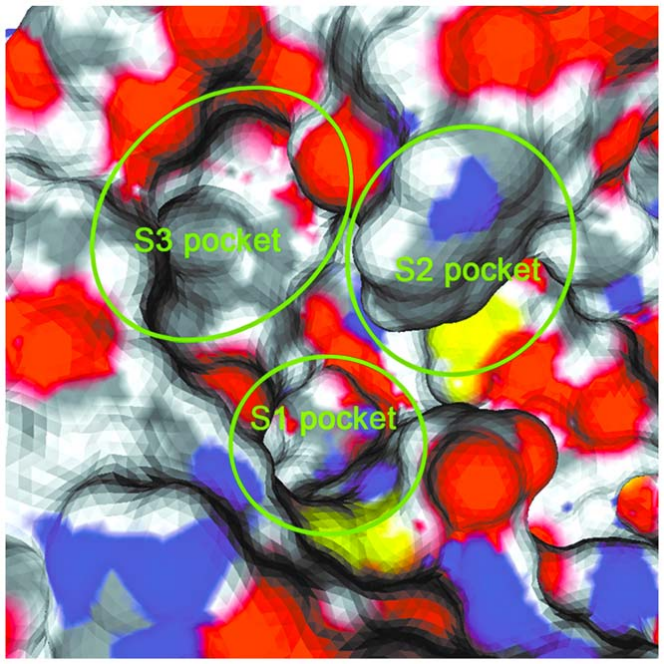
The thrombin active site surface and the pockets of the active site (Sn). The blue color corresponds to nitrogen atoms, red to oxygen atoms, yellow to sulfur atoms, dark grey to carbon atoms and light grey to hydrogen atoms.

**Figure 2:**
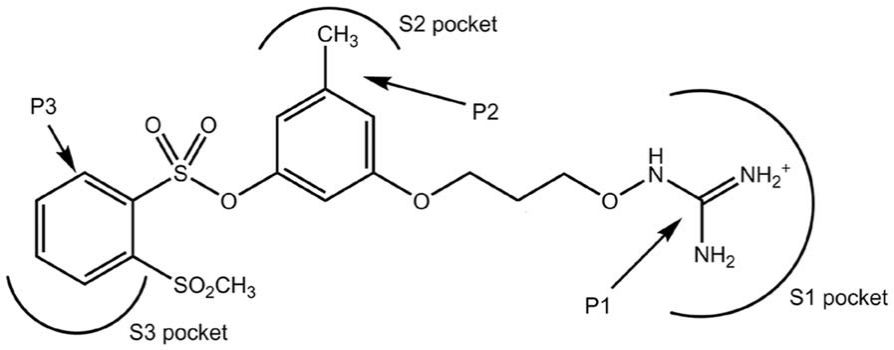
Scheme of disposition of ligand moieties (Pn) in the thrombin active site. One of the known orcinol-based thrombin inhibitors [7] is presented. The active site pockets (Sn) are indicated.

The thrombin active site has three pockets (Fig. 1). The negatively charged residue of aspartic acid (Asp 189) is situated on the bottom of the deep and narrow pocket S1. The two others pockets, S2 and S3, have hydrophobic surfaces. The S2 pocket contains proline and glycine residues, while Leu99, Ile174, and Trp215 residues are situated in the S3 pocket. The S3 pocket binds predominantly to aromatic residues of substrates (or inhibitors) and is thus referred to as the aryl-binding site. Usually, the inhibitor's moieties, located in each of the enzyme active site pockets, are denoted P1, P2 and P3, according to the pocket number. A scheme showing the disposition of inhibitor residues in the thrombin active site is depicted in Fig. 2, using the example of the well-known orcinol-based thrombin inhibitor, which is very similar to new inhibitors developed in this study. This scheme is based on data from the X-ray structure analysis (PDB, code 1T4U) [7].

Virtual screening by means of ligand docking is widely recognized as a helpful approach in modern drug design. We performed computer-aided molecular design employing our own docking program and used the strategy of stepwise experimental screening for the estimation of antithrombin and anticoagulant activities of the compounds with the best scoring functions. Important characteristics, such as acute toxicity and the stability of new inhibitors during long-term storage, were also determined. This combined approach allowed us to shorten the first phase of the search for new thrombin inhibitors and to develop for a period less than 1 year new effective and safe promising drug candidates for medical applications.

## Materials and Methods

### 1. Materials

Donor blood was collected at the Blood Transfusion Station of the National Research Center for Hematology and was used without identifiers. Blood was collected into 3.8% sodium citrate (pH 5.5) at a blood/anticoagulant ratio of 9/1. Platelet poor plasma (PPP) for endogenous thrombin potential measurements was prepared by centrifuging blood for 15 min at 1500 g.

Human thrombin (8.3 mg/ml) was obtained from Hematologic Technologies Inc. (Essex Junction, VT, USA). The thrombin-specific chromogenic substrate Tosyl-Gly-Pro-Arg-p-nitroanilide (identical to Chromozym-TH) was purchased from Sigma (St. Louis, MO, USA). Thrombin-specific fast (BOC-Ala-Pro-Arg-AMC) and slow (BOC-Ile-Gly-Arg-AMC) fluorogenic substrates [8], where BOC is the *tert*-butoxycarbonyl residue and AMC is the 7-amino-4-methylcoumarine residue, were synthesized in the Institute for Medical and Biological Chemistry (Moscow, Russia). Cremophore EL (macrogol-36-glycerol-ricinoleat) was purchased from Caelo (Caesar & Loretz GmbH, Hilden, Germany), and standard normal saline was purchased from Juno, a medical holding company (Medsynthesis Ltd., Novouralsk, Russia). All other domestic chemicals of reagent and analytical grade were used without further purification.

### 2. Methods

#### 2.1. A computer-based search for new thrombin inhibitors

The docking studies were performed using our docking package SOL software implemented in the program complex KeenBASE [9]. The main features of this package are as follows: a rigid target-protein structure, with the active site represented by a set of grids for different type of potentials to describe protein-ligand interactions (electrostatic and Van der Waals (VdW) forces) in the frame of the Merck Molecular Force Field (MMFF94) [10]; a rigorous description of solvation and desolvation effects upon ligand binding, based on the generalized Born approximation [11], [12], which is included in the set of potential grids; a genetic algorithm for global optimization of protein-ligand interaction energy and calculation of ligand internal strain energy in terms of MMFF94.

To perform docking with the SOL program for ligands of any protein, the preprocessing of protein and respective ligands has to be carried out. Usually there are incomplete side chains and missing amino acid residues in structure of the protein in the vicinity of the binding site. In our study missing protein hydrogen atoms were added with the Reduce program [13]. All water molecules, inhibitors and small residues, like sulfates and phosphates, were removed from the complexes. Next, all protein atoms were typified in accordance with the Merck Molecular Force Field (MMFF94) [10] using our own procedure [14]. The docking area was represented by a cube with a 22 Å edge covering the protein active site. The cube center was chosen as the geometrical centre of the native ligand of the respective PDB protein-ligand complex, and the protein structures were saved to mrk files that were suitable for subsequent 101×101×101 grid generation.

The grid of potentials representing thrombin-ligand interactions was calculated separately using the SOL_GRID program [9], before the initiation of the docking procedure. Throughout the docking studies, all ligands were considered fully flexible – i.e., all topologically available torsional degrees of freedom were unfrozen and allowed to rotate freely, directed only by ligand internal energy preferences in the frame of MMFF94. Bond lengths and valence angles were frozen in the course of the docking procedure.

The careful validation of the SOL docking program was carried out using two different validation protocols to test the correctness of the physical and mathematical principals implemented in this docking program [9], [15]. The first protocol concerns the identification of active ligands among a mixed set of active and inactive ones. The second protocol [16] concerns the determination of accuracy for positioning ligands in proteins active sites. This protocol was used to compare the two docking programs, SOL and the standard AutoDock 3.05. The first protocol showed a good to excellent quality in the SOL program for the selection of active inhibitors for four different target-enzymes from a large set of active and inactive (“rubbish”) ligands [9]. The accuracy of ligand positioning in the active sites of enzymes was defined by the root mean square deviation (RMSD) between ligand docked poses and experimental ligand poses taken from the Protein Data Bank [17]. The results of the docking quality comparison for both programs demonstrated that the docking quality of SOL is better than that of AutoDock 3.05, if we consider docking quality with the criterion RMSD<1.5 Å. Almost twice as many native ligands docked by SOL had a RMSD≤1 Å when compared to the respective number of ligands docked by AutoDock 3.05 [9].

The thrombin 3D structure was taken from the Protein Data Bank (PDB, code 1O2G). All possible ligand poses within 22 cubic angstroms around the center of the thrombin active site were considered in docking. Electrostatic, VdW and solvation-desolvation potentials were calculated on a 101×101×101 grid inside this cube. Parameters of the genetic algorithm (population size 30000, mating pool size 70, number of generation 300, number of runs 50, and parameters for definition of elitism, mutation, crossover, niching) [9] were chosen to get the best docking results for the native ligand of the 1O2G PDB complex and for the thrombin-argatroban complex (1DWC PDB complex) with an accuracy of 1.5 Å.

Three-dimensional structures of ligands (thrombin inhibitor candidates) for initial virtual screening experiments with compounds received from the National Cancer Institute (NCI) were taken directly from the NCI Diversity set of compounds [18]. The next steps of the virtual screening were performed with our specially designed virtual ligand libraries. 3D structures of ligands constructed during the hit optimization process were generated by means of the CORINA 3D structure generation service [19]. Virtual screening was performed using a massive-parallel supercomputer using X-Com grid technology, developed at the Research Computer Center of Moscow State University [20], [21]. Visual inspection of ligand poses within the thrombin active site, depicted as Solvent Excluded Surfaces (SES), was performed with the help of the TAGSS program for triangulated SES construction and visualization [22].

#### 2.2. Measurement of thrombin induced hydrolysis of specific substrates

The kinetics of thrombin inhibition was determined from the hydrolysis reaction of a specific substrate by thrombin in the presence of the tested substances. The chromogenic substrate (Tosyl-Gly-Pro-Arg-p-nitroanilide) or fast fluorogenic substrate (BOC-Ala-Pro-Arg-AMC) was used.

Plate wells were filled with 20 mM HEPES (pH 8.0) containing 140 mM NaCl and 0.1% polyethylene glycol (molecular weight 6000 Da). Thereafter, substrate was sequentially added to each well (final concentration, 100 µM), followed by the substance being tested (to a final concentration that was varied) and thrombin (final concentration, 0.2 nM). The hydrolysis rate was monitored spectrophotometrically at 405 nm (absorption maximum of the reaction product – *p*-nitroaniline), or fluorometrically (λ_ex_ = 380 nm; λ_em_ = 440 nm for fluorescent reaction product 7-amino-4-methylcoumarine).

The initial rate was determined as the slope of the linear portion of the kinetic curve over the first 10 to 20 min of measurement. The inhibitory effect was expressed as the percentage reduction of the initial hydrolysis rate. The reaction rate in the absence of inhibitor was taken as 100%. Each result is the mean of two parallel determinations.

The inhibitor concentration was determined decreasing hydrolysis rate to 50% (IC_50_). Values of the inhibition constants were calculated on the assumption about competitive mechanism of inhibition using equation (1)
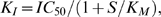
(1)where: *K_I_* - the constant of inhibition,

*S* - the concentration of a substrate (100 µM),
*K_M_* - the Michaelis constant of a specific substrate for thrombin (9.44 µM, average from ref. [23], [24] and our measurements for chromogenic and 13 µM for the fluorogenic substrate [8], respectively).

#### 2.3. Thrombin generation test

The kinetics of thrombin generation in plasma was monitored using a modification of the standard method [25] using a slow fluorogenic substrate BOC-Ile-Gly-Arg-AMC [8]. Normal donor plasma (PPP) was placed in the wells (90 µl/well) of a 96-well flat-bottom microtiter plate, after which 10 µl of the substance solution to be tested was added in different concentrations, along with 10 µl of the slow fluorogenic substrate (initial concentration 5 mM). In the control wells (without inhibitor), 10 µl of the solvent used was added (20 mM HEPES, pH 7.5 and 140 mM NaCl; or pure ethanol, or dimethylsulfoxide (DMSO) for different substances). The samples were incubated at 37°C for 3–5 min before being activated with thromboplastin, which was a 1∶250 dilution of a standard prothrombin time reagent (Renam, Moscow, Russia) in the same buffer supplemented with 90 mM CaCl_2_. The final concentration of tissue factor during activation was about 4 pM. Thromboplastin (25 µl) was added to all wells simultaneously using a multichannel micropipette. The moment of thromboplastin addition was taken as time zero. The kinetics of accumulation of the fluorescent reaction product 7-amino-4-methylcoumarine (AMC) was recorded for 65 min with a fluorometric Fluoroscan II reader (LabSystem, Finland) (λ_ex._ = 380 nm; λ_em._ = 440 nm). The results were averaged over two parallel samples. The error of parallel measurements was within 2–5%. At every moment, the rate of product accumulation is proportional to the instant thrombin concentration present in plasma. Therefore, the curve for the temporal evolution of thrombin in the system may be obtained using differentiation of the curve for AMC accumulation. For each sample, arbitrary fluorescence units were converted to absolute AMC concentrations using the calibration signal obtained by adding a known AMC concentration into each particular plasma sample in the presence of the fluorogenic substrate and the tested substance. The corrections were done taking into account the substrate consumption and the nonlinearity of the AMC calibration curve. The area under the thrombin time curve over a period of 60 min (endogenous thrombin potential, ETP) was determined.

The standard method for the measurement of thrombin generation assumes plasma sample dilution. We have shown before [26] that the ETP value depends on the level of dilution. Because it is especially important for the study of hemodilution, we have developed a modification of the thrombin generation test that excludes additional dilution of the samples during measurement.

In particular, 200 µl of PPP samples were placed in the wells of a 96-well plate. Then, 2 µl of a solution of fluorogenic substrate BOC-Ile-Gly-Arg-AMC in DMSO (initial concentration 41 mM) was added, and coagulation was triggered by 3 µl of activator, which was a 1∶20 dilution of a standard prothrombin time reagent (Renam, Moscow, Russia) in the buffer (20 mM HEPES, 140 mM NaCl, pH 7.5) supplemented with 1.4 M CaCl_2_. Measurement of fluorescence (120 min) and processing of the results obtained were carried out by the methods described earlier.

#### 2.4. Experimental study of the new thrombin inhibitor 4i in vivo using model of hemodilution-induced hypercoagulation in rats

The study was approved by the Ethics Committee of the Center for Theoretical Problems of Physicochemical Pharmacology (Permit Number: 21-04-2009). The housing and operating conditions for animals strictly satisfied the requirements of the Guide for Care and Use of the Laboratory Animals [27]. All efforts were made to minimize suffering in animals.

The anticoagulation activity of one of the new thrombin inhibitors (**4i**) was studied *in vivo* in a newly developed model of hemodilution-induced hypercoagulation in rats. Experiments were carried out on male outbred white rats weighing 250–450 g and housed in a vivarium. A polyethylene cannula was implanted into the right femoral artery of the rats, which were anesthetized with thiopental (40 mg/kg of body weight, i.p.), to a depth of 4–5 cm. Blood loss (23.0±4.5% of estimated blood volume) and hemodilution with plasma-substituting solution (PSS) were simulated by fast blood sampling (4.5 ml during approximately 1 min) and bolus administration of an equal volume of either normal saline (control group) or normal saline supplemented with 2 µM of the thrombin inhibitor (experimental group). Coagulation was analyzed in samples of blood collected before hemodilution for all animals. Blood samples for repeated analysis were taken from each animal only once at 10, 30 or 60 min after the PSS infusion. The coagulation system status before and after PSS infusion was investigated by endogenous thrombin potential measurement. The volume of blood loss was calculated on the assumption that the total blood volume of the rat is equal to 6.5% of the body weight.

#### 2.5. Measurement of the acute toxicity of new compounds in mice

Preliminary safety examination for the best synthesized inhibitors was carried out by measuring their acute toxicity in C57Bl/6 mice (females, 26–28 g, intraperitoneal administration) [28]. The total number of animals used in each experiment was 20–35. The dose of inhibitor that caused mortality in half of the animals after 2 or less hours (LD_50_) was determined. The solution of inhibitor (initial concentrations ranged from 50 to 7 mg/ml depending on substance solubility) in normal saline supplemented with 20% of cremophore EL to improve the inhibitor's solubility was administered to animals intraperitoneally. For administration of different doses of an inhibitor, different volumes of the initial stock inhibitor solution were supplemented with corresponding volumes of vehicle (20% cremophore EL in normal saline) to obtain a constant administered volume of 1.8 ml in each case. To obtain the highest doses of inhibitors, the animals were injected with 2.3 ml of stock inhibitor solutions instead. Preliminary results showed that after the administration of 2.3 ml of normal saline supplemented with 20% cremophore EL, animals did not have any toxic manifestations.

#### 2.6. Stability of inhibitors in solution

Activities of the best new thrombin inhibitors were measured by the inhibition of chromogenic substrate hydrolysis (see section 2.2) during long-term storage in physiological saline (1 µM solutions) at room temperature and at 4°C. All solutions were sterilized before storage by autoclaving (120°C, 1 atm, 20 min).

## Results

### 1. Design of new thrombin inhibitors

**Figure 3:**
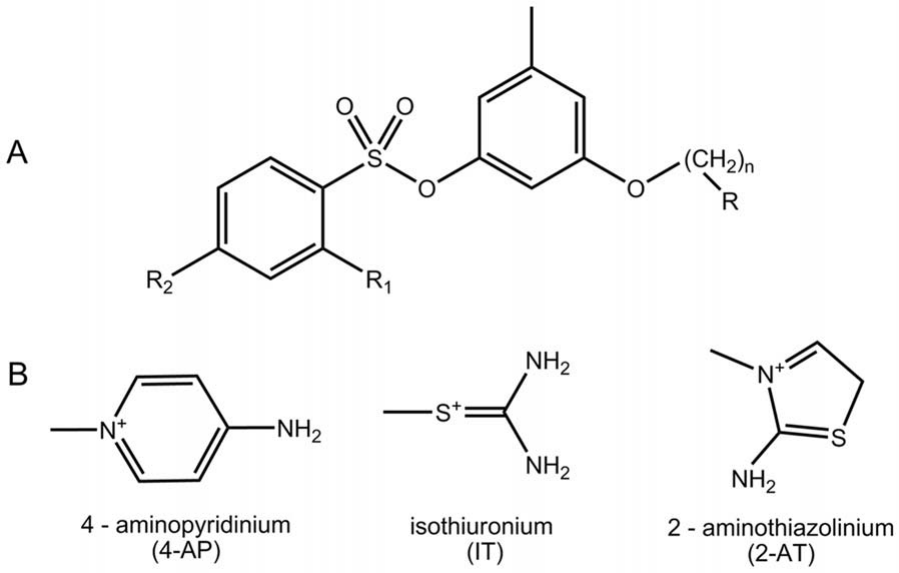
The newly developed thrombin inhibitors. (A) – The common structural formula of new compounds. (B) – The most effective fragments P1 obtained in this study.

Nonpeptide thrombin inhibitors based on an orcinol scaffold have been described in the literature. In spite of their structural simplicity, these inhibitors are relatively effective (K_I_ values are in the order of several tens of nM) and have sufficient selectivity towards thrombin [29]–[32]. In this study, we tried to improve the potency of such inhibitors. We used traditional orcinol core and benzenesulfonic acid residues as the P2 and P3 fragments of the inhibitor molecules but varied the basic P1 moiety and the length of the linker between the P1 moiety and the aromatic P2 fragment of the compounds. In addition, we examined compounds with different substituents in the benzenesulfonic acid fragment (R1 and R2 in P3). The basic structure of the new compounds is presented in Fig. 3A.

**Figure 4:**
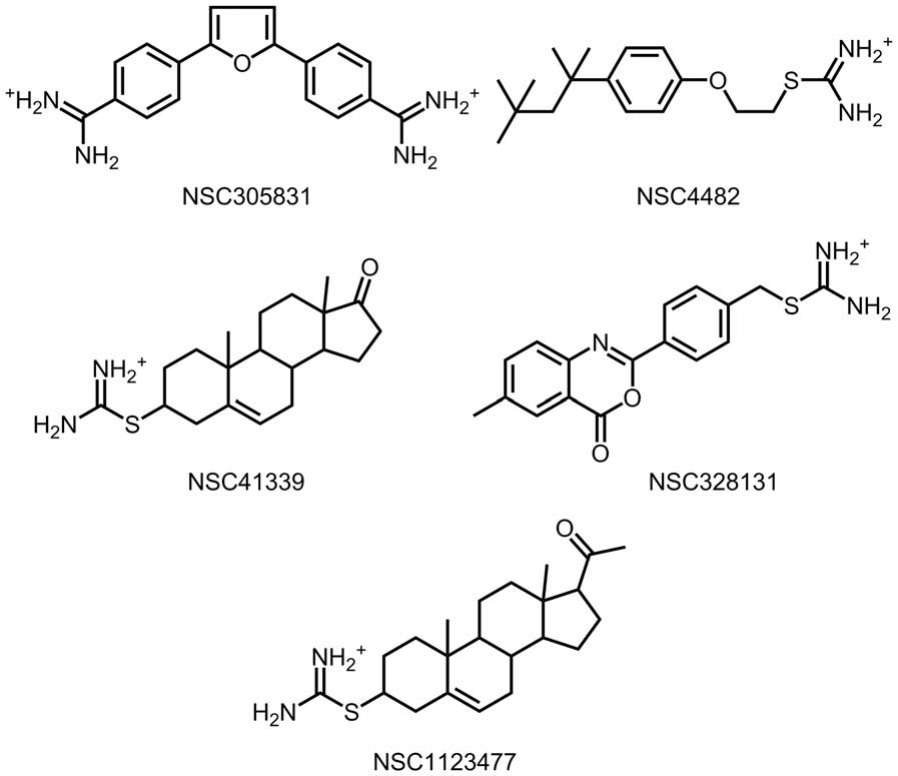
Structures of the five best compounds received from NCI. NCI ID numbers are indicated.

The first step of our search for new P1 moieties was a virtual screening of the existing positively charged ligands from the NCI/DTP Open Chemical Repository (National Cancer Institute, USA) [18], including the whole of the NCI Diversity Set [33]. Compounds from the NCI library were used in the very beginning of the study to try out and debug a docking program and to adjust it to thrombin. Given that those compounds had already been synthesized by that time and were kindly gifted to us by the NCI, we had an opportunity of not only calculating their scoring functions with our docking program, but also measuring their real (not virtual) inhibitory potencies experimentally. More than 2000 compounds were virtually screened and as a result 114 virtual lead substances were selected for subsequent experimental verification. These compounds were obtained from NCI. The criteria for the hits selection were sufficiently negative SOL scoring function (represented as predicted protein-ligand binding energy <−5.5 kcal/mol) and reasonable ligand poses in the thrombin active site, verified by visual inspection. Among these 114 compounds, 15 proved to be real thrombin inhibitors in our experimental tests with specific thrombin substrates. Five compounds had sufficiently high activities to be interesting for subsequent drug design. Four of these compounds possessed an isothiuronium group in the P1 position of the molecule, which has never been used as a P1 moiety in a thrombin inhibitor molecule before (Fig. 4 and Table 1). The high activity and reduced basicity of isothiouronium *vs.* traditional guanidinium derivatives make the isothiouronium residue an interesting possibility for the design of new thrombin inhibitors.

**Table 1:** The SOL scoring functions and the results of experimental measurements of thrombin inhibitory activity for the five best compounds received from NCI (see Fig. 4).

NCI ID	SOL scoring function, kcal/mol	Inhibition of specific thrombin substrate hydrolysis in buffer system	
		IC_50_, µM (substrate used)1)	K_I_, µM2)
**NSC 305831**	−6.14	72 (S_CHR_)	6.2
**NSC 4482**	−6.21	3.45 (S_FLUO_)	0.4
**NSC 41339**	−6.25	240 (S_FLUO_)	27.6
**NSC 328131**	−6.39	∼353) (S_FLUO_)	4.0
**NSC 123477**	−6.41	75 (S_FLUO_)	8.6

1) The chromogenic (S_CHR_), or fast fluorogenic (S_FLUO_) specific thrombin substrate was used.

2) Values of K_I_ were calculated assuming a competitive mechanism of inhibition.

3) This substance has slow inhibition kinetics. The K_I_ value was calculated using the end point of the inhibition for each concentration of inhibitor.

Taking into account the data in the literature and the results obtained during computer-based and experimental screening of ligands from NCI, we attempted to construct new chemical structures that should be effective thrombin inhibitors. As mentioned before, the known residues of orcinol and benzenesulfonic acid were selected as P2 and P3 inhibitor fragments, respectively, but different basic P1 fragments were examined.

Computer-based screening of more than 6000 compounds gave evidence of possible inhibitory compounds with 4-aminopyridinium (4-AP), isothiuronium (IT) or 2-aminothiazolinium (2-AT) fragments in the P1 position of the ligands (Fig. 3B). The SOL scoring functions of many of these compounds were sufficiently negative. The last type of moiety that was studied was the “reversed” and constrained analog of isothiouronium. The examples of the scoring functions for some compounds with different P1 fragments are presented in Table S1.

Theoretical calculation showed that an increase in the linker length between the P1 and P2 fragments in molecules of IT and 4-AP derivatives should give rise to a less negative scoring function. This is clearly illustrated in Table 2 for a series of 4-AP compounds with different linker lengths. Introduction of different R1 and R2 substitutes in the aromatic ring of the P3 fragment modified the scoring function, sometimes noticeably, but we could not identify any regularity in these changes (Table S2).

**Table 2:** Examples of scoring function values for compounds with constant a P1 part, but a different length of the linker (n) between the P1 and P2 fragments of a molecule1).

The length of the linker (n)	1	2	3	4	5
**Scoring function, kcal/mol**	−7.50	−6.57	−6.48	−6.27	−5.45

1) The common formula for these compounds is depicted in Fig. 3A. R1 and R2 are H, R (in P1 position) is residue of 4-AP.

### 2. The experimental examination of new compounds

**Figure 5:**
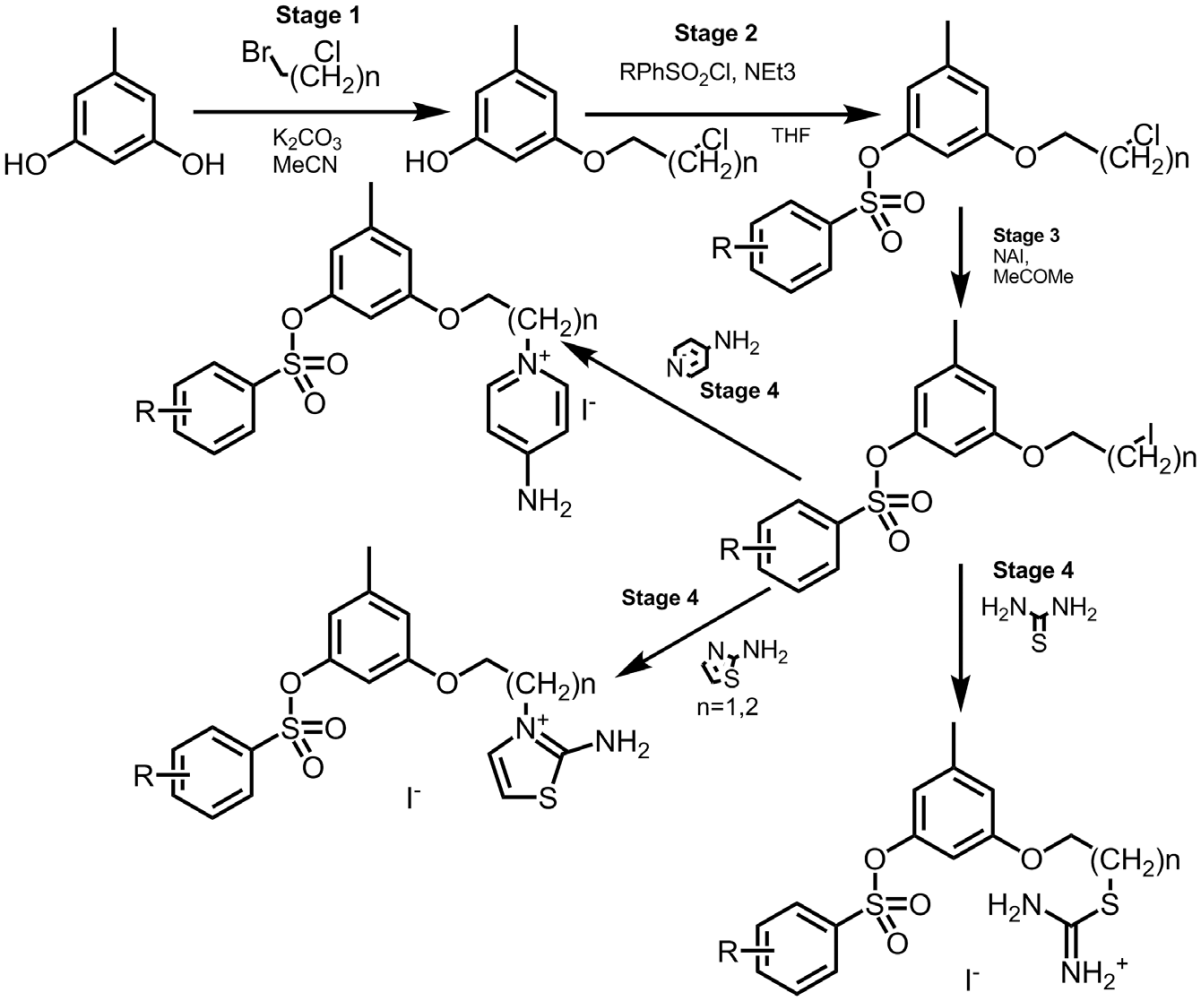
Synthetic scheme of the new orcinol-based thrombin inhibitors. Reagents: (*stage 1*) BrCH_2_(CH_2_)_n_Cl, K_2_CO_3_, (CH_3_)_2_CO or CH_3_CN, reflux, 36–43 h; components of reaction mixture were used for subsequent reactions without purification of individual ingredients; (*stage 2*) RPhSO_2_Cl, Et_3_N, THF, stirring, 20–25°C, 7 h or ArSO_2_Cl, PyH, 20–25°C, 3 days then 60°C, 2 h; (*stage 3*) NaI, (CH_3_)_2_CO or CH_3_CN, reflux, 48 h; (*stage 4*) corresponding nucleophile dioxane, 80°C, 2 days or reflux, 20 h.

Some of the aforementioned new compounds were synthesized on a 0.25–2.5 g scale via a 2 to 4 step strategy. The schemes of the compounds' syntheses are depicted in Fig. 5. Details of these syntheses are described in [34] and in the Text S1. All compounds have been numbered according to the course of preparation. Compounds **1a–b**, **2a–j**, and **3a–j** served as intermediates in the syntheses of the final inhibitors. These compounds are presented only in the descriptions of the Text S1. As Table 3 contains only the most promising new inhibitors, some other final compounds (inhibitors **5**, **6**, and **7a–d**) are described only in the Text S1 and are presented in Table S2. The chemical structures of all new inhibitors were confirmed by ^1^H-NMR data (see the Text S1).

**Table 3:** Parameters of thrombin inhibition and acute toxicity for the best new thrombin inhibitors and argatroban1).

N2)	R	R1	R2	K_I_ for thrombin inhibition in buffer system^3^^,^ 4), nM	IC_50_ for reduction of thrombin generation in plasma, µM	LD_50_ (mouse), intraperitoneal administration, mg/kg
**4e**	4-AP	-F	-H	0.3	0.1	523.1±92.3
**4f**	4-AP	-Cl	-H	0.21	0.26	679.0±152.7
**4g**	4-AP	-SO_2_CH_3_	-H	0.75	0.16	166.7±20.0
**4h**	4-AP	-H	-Cl	0.78	0.15	372.7±84.6
**4i**	4-AP	-H	-H	0.78	0.25	418.9±33.0
**8b**	IT	-SO_2_CH_3_	-H	1.5	0.80	>458.05)
**8c**	IT	-Cl	-H	0.33	0.16	793.5±118.5
**8d**	IT	-H	-H	0.95	0.90	>1111.1
**8f**	IT	-F	-H	2.03	1.24	>653.15)
**8h**	H_3_C-NH-IT6)	-Cl	-H	5.7	0.53	>287.05)
**Argatroban** 7)				39	0.65	475

1) The common formula for all inhibitors is depicted in Fig. 3A.

2) Synthesis details are in the Supporting Information.

3) Measured by chromogenic substrate Tosyl-Gly-Pro-Arg-p-nitroanilide.

4) The K_I_ values were calculated with the assumption of a competitive type of inhibition. The K_M_ value of the substrate for thrombin is 9.44 µM.

5) Substance is nontoxic in thousand-fold ETP reduction IC_50_ dose (per whole mouse).

6) IT contains methyl group as a substituent in one of the amino groups.

7) All constants for argatroban (except for the LD_50_) were measured and were in good agreement with data in the literature [35]–[38].

For all new compounds, the antithrombin activity in buffer systems *in vitro* and the anticoagulant activity in plasma *in vitro* were determined using the thrombin generation test. One of these new inhibitors (**4i**) was investigated in an *in vivo* rat model of hemodilution-induced hypercoagulation.

### 3. Inhibitory activity of new substances towards thrombin in buffer system

**Figure 6:**
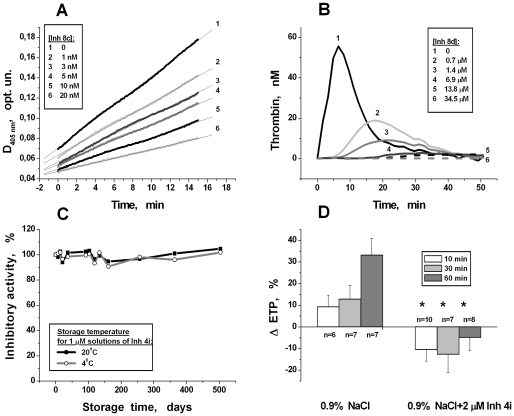
Examples of some typical results obtained during experimental testing of new inhibitors. (A) – Inhibition of thrombin-induced chromogenic substrate hydrolysis in buffer systems by different concentrations of **8c**. (B) – Reduction of endogenous thrombin potential in plasma by increasing concentrations of **8d**. (C) – Stability of thrombin inhibitory activity of **4i** (1 µM) during long-term storage in physiological solution at 4°C or at room temperature. (D) – Influence of physiological solution (control group) or of this PSS supplemented with 2 µM of **4i** (experimental group) on the development of hypercoagulation in models of hemodilution-induced hypercoagulation in rats. The changes of the ETP values are presented relatively to basic level in different times after PSS infusions. * - The differences were considered as statistically significant (*P<0.05*, Student's t-test, n = 6–10).

All new compounds inhibited the hydrolysis of the specific substrate by thrombin in a dose-dependent manner, achieving total inhibition at some concentrations (Fig. 6A). Similar patterns of behavior are usually typical for competitive inhibitors. Table 3 presents the constants of thrombin inhibition for the best of the newly developed inhibitors. These constants were calculated from the measured IC_50_ values with the assumption of a competitive type of inhibition. Data for argatroban are presented in Table 3 for comparison [35]. Similar data for other newly synthesized compounds are presented in Table S2. As a result, several compounds of IT, and especially of the 4-AP series, were shown to be the most active orcinol-based thrombin inhibitors. Some of these compounds were the most active from known low molecular weight DTIs with K_I_ in the sub-nanomolar range.

### 4. Anticoagulant activity of new inhibitors in plasma *in vitro*

Anticoagulant activity of an inhibitor in plasma is dependent not only on its constant of inhibition, but also on possible interactions with other components of the coagulation system and on binding with plasma proteins, especially with albumin. Therefore, in the next step of experimental testing, we examined in plasma *in vitro* the anticoagulant activity of the effective in buffer system new compounds. It was shown by the thrombin generation test that these compounds reduced ETP in plasma. The value of reduction increased with an increasing concentration of the inhibitor (Fig. 6B). Additional concentrations that decreased ETP by 50% (IC_50_ for ETP reduction) are also presented in Tables 3 and S2.

Although the IC_50(ETP)_/K_I_ ratio for new compounds was high, the best compounds had IC_50_ values for ETP reduction that were below the corresponding value seen in argatroban (0.6–0.97 µM) [36], [37]. Thus, the best of the designed compounds could have great potential for *in vivo* studies and subsequent medical applications in the injection form.

### 5. Acute toxicity of new thrombin inhibitors

A preliminary study of the best newly synthesized inhibitors acute toxicity was performed, and the safety of these compounds was evaluated *in vivo*. The results obtained are presented in Table 3. The acute toxicity (LD_50_) in mice for several of the new inhibitors proved to be lower than that for clinically used argatroban [38] (Table 3).

### 6. Stability of the inhibitors in aqueous solutions

All new inhibitors selected for long-term storage were stable after sterilization by autoclaving. The activities of the most effective new thrombin inhibitors remained stable after storage in physiological saline (1 µM solution) over more than 1.5 years, both at room temperature and at 4°C. As an example, activities of compound **4i** during long-term storage are presented in Fig. 6C.

### 7. Model of hemodilution-induced hypercoagulation in rats

The rat hemodilution-induced hypercoagulation model was developed for testing the effectiveness of new inhibitors *in vivo*. Previously we have shown that plasma dilution with crystalloid solutions *in vitro* intensified coagulation, whereas the addition of a thrombin inhibitor to the solution partially neutralized that effect [26], [39]. In this work, we applied the same approach to hemodilution *in vivo*. Animals with 23.0±4.5% blood loss were infused with an equal volume of either standard saline (control group) or of saline supplemented with 2 µM of the new inhibitor **4i** (experimental group). The changes of the endogenous thrombin potential (ETP) with respect to its initial level for the control and experimental groups at different times after infusion are presented in Fig. 5D. The results obtained showed that animals infused with normal saline developed hypercoagulation that increased with time, whereas the presence of the thrombin inhibitor in the PSS canceled the hypercoagulation effect of hemodilution in the experimental group.

## Discussion

A fast decrease of preformed thrombin activity rises is vital in acute situations. Thus, it is reasonable in such cases to intravenously administer direct thrombin inhibitors to block hypercoagulation as quickly as possible. Our aim was to design new thrombin inhibitors for intravenous administration, whereby inhibitors can get directly to blood plasma where thrombin works. Thus, bioavailability was not an issue, and we were not restricted to ligands with low basicity in their P1 fragments.

We have shown before that moderate plasma dilution *in vitro* (up to 2–2.5 times) with different artificial PSS produced hypercoagulation changes in the coagulation system (the ETP and spatial clot growth rate were increased) [26], [39]. This fact suggests that plasma dilution, especially by crystalloid PSS, could also be a risk factor for the induction of thrombotic states during moderate hemodilution *in vivo*. The development of hypercoagulation has been shown to correlate with the infusion of large volumes of crystalloid solutions in patients [1], [2]. At present, the mechanism of this phenomenon is not clear; however, many investigators propose that during moderate hemodilution, the coagulation system is more sensitive to decreasing concentrations of coagulation inhibitors than to dilution of procoagulant factor precursors that are present in the blood in abundance.

To prevent the development of hemodilutional hypercoagulation, we supplemented a crystalloid PSS with DTI [26], [39]. It was shown that the natural thrombin inhibitor antithrombin III could be used for this purpose [39]. However, this inhibitor is isolated from human plasma and is thus very expensive and not completely safe with regard to the transmission of viral infections. Small molecule synthetic thrombin inhibitors are more suitable for this purpose. To be used in PSS, these inhibitors should be not only highly effective and safe, but also stable in aqueous solutions. The development of this kind of inhibitor was one of the objectives of our study.

A majority of successful thrombin inhibitors have positively charged or neutral but easy polarizable (e.g. containing halogen atoms) P1 fragments (Fig. 2). During thrombin-inhibitor complex formation, the P1 moiety of the inhibitor is located in the thrombin active site within a narrow cavity, exposing the carboxyl side chain of the Asp189 residue on its bottom (S1 pocket in Fig. 1 and Fig. 2).

The severe spatial restrictions dictate the small size and hydrophobic nature of the P2 inhibitor position. In contrast, the restrictions on the P3 site are not as stringent because the corresponding binding site in the thrombin molecule is broad and exposed to the solvent. This feature gives also us the opportunity to modify the part of the P3 moiety, which is projected into the solvent, to increase the hydrophilic nature of the inhibitor and modify, for example, its solubility and lipophilicity characteristics.

The selection of effective ligands for the inhibition of a target enzyme is usually a very laborious, long and expensive process. Computer-aided screening using well adjusted docking program allowed us to shorten this stage of the study. Adjustment of our program, SOL, for the thrombin inhibitor search was executed during a screening of the NCI database, because we compared actual inhibitory activities of these compounds to their scoring functions in our theoretical calculations. As a result, five new inhibitor molecules were discovered. Besides, while screening compounds from NCI, we discovered that some compounds with an isothiuronium group in the P1 position of the ligand were sufficiently effective thrombin inhibitors. Currently, this moiety has not been used as a fragment in the P1 position of thrombin inhibitors.

**Figure 7:**
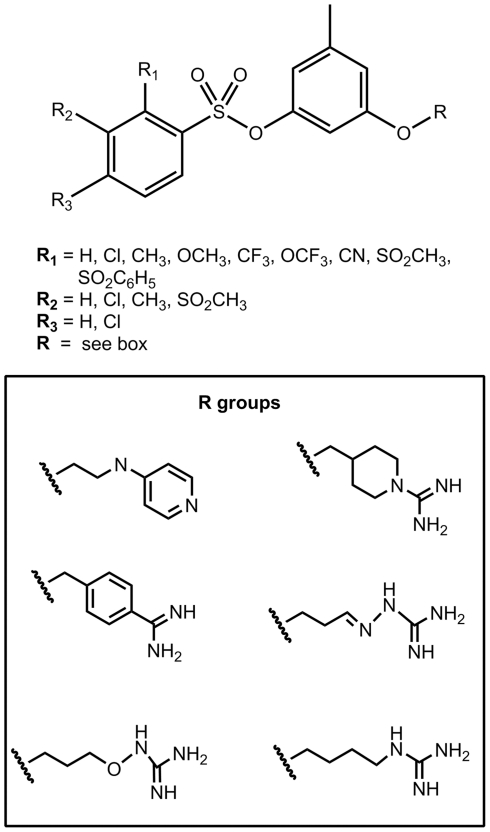
The known orcinol-based thrombin inhibitors described in the literature [**29]**–[**32**].

In the next stage of the study, we generated large virtual libraries of ligands as possible thrombin inhibitors, taking into account all discovered patterns. We focused on variations of basic fragments in the P1 position and on a search for the optimal linker length connecting this fragment with the residue in the P2 position of inhibitor. As was mentioned before, the orcinol and benzenesulfonic acid residues were selected as P2 and P3 fragments of our new inhibitors, respectively. Existing inhibitors containing these fragments are pictured in Fig. 7 [29]–[32]. These inhibitors were selected for modification because they are relatively simple, sufficiently effective (K_I_ in the nM range) and highly selective. The overall number of compounds studied in virtual screening experiments was near 6000. These calculations have shown that the introduction of a 4-aminopyridinium (4-AP), isothiuronium (IT), or 2-aminothiasolinium (2-AT) group in the P1 position of the compound (Fig. 3B) should give rise to a high inhibitory activity. According to these calculations, the inhibitory effectiveness should improve when the length of the linker between the P1 and P2 fragments of the inhibitor molecule decreases from 5 to 1 CH_2_ groups (Table 2).

Several series of new compounds were synthesized to experimentally confirm the accuracy of these theoretical conclusions. Their inhibitory activity was first checked in different experimental procedures *in vitro*.

The direct antithrombin activity of new compounds was confirmed by the measurement of the inhibitory effect on the hydrolysis rate of specific chromogenic substrate in the presence of a constant concentration of thrombin in a buffer system. All of the synthesized compounds decreased substrate hydrolysis rate in a dose-dependent manner. The results of one of such experiment are presented in Fig. 6A. However, antithrombin activity is insufficient to make these new compounds actual anticoagulants in plasma. The anticoagulation activity of an inhibitor in plasma is dependent not only on its inhibition constant but also on possible interactions with other components of the coagulation system and on binding with plasma proteins, especially albumin. We used a thrombin generation test for the characterization of coagulation in plasma. Endogenous thrombin potential (ETP) is one of the parameters of this test. ETP is the total quantity of active thrombin arising in plasma after standard coagulation activation. It is equal to the area under the thrombin kinetic curve. The presence of additional thrombin inhibitors in the plasma sample should change the thrombin formation and inhibition kinetics. As a result, ETP should decrease. All new inhibitors significantly decreased ETP. The effect value also increased with an increasing concentration of the inhibitor, and at some concentrations, thrombin generation was completely inhibited (Fig. 6B).

Thus, the results obtained show that these new compounds are effective thrombin inhibitors and have high anticoagulant activity in plasma *in vitro*. Moreover, these inhibitors excellently retain activity after long-term storage in aqueous solutions (Fig. 6C). For the best new compounds, the effectiveness (K_I_<1 nM; minimal IC_50_ for ETP reduction, 0.1 µM) and stability in aqueous solutions was better than for argatroban (Tables 3 and S2).

Experimental screening showed that our inhibitors with new P1 fragments were highly effective. Inhibitory efficacy was much greater for compounds with a linker length of n = 2 as compared to n = 3. The SOL scoring function correctly estimated that 4-AP and IT derivatives with a 2 carbon chain linker between the basic P1 group and the orcinol core (n = 2 in Tables 3 and S2) should be more potent than the derivatives with a 3 carbon chain linker (n = 3), although the magnitude of this difference is underestimated by the SOL score (Table S2). Because of the small number of 2-AT derivatives synthesized, we do not present a similar dependence for these compounds.

Theoretical calculations predicted sufficient differences in scoring functions for compounds with different R1 and R2 substituents in the P3 fragment of inhibitor molecule. In spite of this, the results obtained showed that, with the exception of the p-CH_3_ substituent, introduction of different substituents in the ring of benzenesulfonic acid had a relatively weak influence on K_I_ and IC_50_ values for ETP reduction (Table S2).

Hence, according to a comparison of the experimental testing results with the theoretical prediction of the power of new inhibitors, we conclude that our docking program is excellent in searching for ligands with an effective basic fragment P1, and it correctly presents the tendency of inhibitor efficacy to change according to linker length. However, it is not suitable for the fine analysis of the effectiveness of structures with different substituents in the benzenesulfonic acid group in the P3 position of a molecule.

The examination of acute toxicity shows that the LD_50_ values of the new inhibitors are comparable, and sometimes even higher, than those seen for the clinically used argatroban (Table 3). In addition, toxic effects appear in doses 2000–5000 times higher than the appropriate therapeutic dose. Also, the new compounds appear to be very stable during long-term storage in aqueous solutions.

After examining the new inhibitors' effectiveness, stability and safety in acute experiments, the anticoagulant efficacy one of the new compounds (**4i**) was also studied *in vivo* in a model of hemodilutional hypercoagulation in rats. It was demonstrated experimentally that the hypercoagulant state has developed *in vivo* after the infusion of a sufficiently large volume of crystalloid PSS. Similar to *in vitro* experiments [26], the introduction of direct thrombin inhibitor in PSS canceled this effect completely (Fig. 6D). The inhibitor selected for these experiments (**4i**) has an IC_50_ value for reduction of ETP *in vitro* equal to 0.25 µM. We supposed that after *in vivo* administration, this inhibitor could be accumulated in different organs and tissues. The inhibitor can be also partially consumed after the initiation of coagulation. Therefore, a 2-µM concentration of the inhibitor was selected for supplementation of PSS in experiments. It is necessary to note that the selected inhibitor concentration turned out to be too high. It should be decreased, if the aim was to return the ETP to the normal initial value. Therefore, this inhibitor was very effective after intravenous administration *in vivo*.

The DTIs that were developed are very suitable for intravenous administration. However, it is obvious that the development of new anticoagulants for peroral introduction is also a very important objective for the amelioration of antithrombotic therapy, especially prophylactic therapy. The main problem of these treatments is low bioavailability of the DTIs using this type of administration. One possible solution of this problem is the development of prodrugs. In these compounds, the active parts of inhibitor molecules are protected by special groups that are removed, leading to formation of the active inhibitor directly in the body after passing through the mucous membrane of the gastrointestinal tract [40]. We suppose that our new inhibitors could be a good basis for the development of such proinhibitors, and their application will not be restricted to only intravenous administration.

The obtained results show that our docking approach, augmented by experimental screening, is a powerful strategy to find new inhibitor motifs and to improve the potency of inhibitors. We developed new effective, stable, and safe thrombin inhibitors. Furthermore, these inhibitors not only slow down coagulation in different tests *in vitro*, but they also prevent the appearance of a hypercoagulant state in models of hemodilutional hypercoagulation in rats *in vivo*. These compounds are very promising, but further detailed studies are necessary to confirm the possibility of medical applications for these new inhibitors.

## Supporting Information

Table S1 Examples of scoring function values for compounds with different R moieties in the P1 position of a molecule^1)^.
(DOC)

Table S2 The new thrombin inhibitors – derivatives of orcinol, their structures, SOL scoring function values and results of experimental testing.
(DOC)

Text S1 Supplementary Materials and Methods.
(DOC)
