# Supplementary material for: New Synthetic Thrombin Inhibitors: Molecular Design and Experimental Verification
Source: PLoS One. 2011 May 16;6(5):e19969. doi: 10.1371/journal.pone.0019969 (PMC3095642; doi:10.1371/journal.pone.0019969)
Supplement: Table S1 — Examples of scoring function values for compounds with different R moieties in the P1 position of a molecule1). (DOC) [file pone.0019969.s001.doc]

**Table S1.** Examples of scoring function values for compounds with different R moieties in the P1 position of a molecule^1)^

| **Fragment R in the P1 position** | **Scoring function, kcal/mol** |
| --- | --- |
|  | -4.52 |
|  | -4.57 |
|  | -5.00 |
|  | -5.33 |
|  | -5.45 |
|  | -5.52 |
|  | -5.58 |
|  | -6.12 |
|  | -6.57 |

^1)^ Common formula for all compounds is presented in Fig. 3A. R1 and R2 are H, n=2
