# Supplementary material for: New Synthetic Thrombin Inhibitors: Molecular Design and Experimental Verification
Source: PLoS One. 2011 May 16;6(5):e19969. doi: 10.1371/journal.pone.0019969 (PMC3095642; doi:10.1371/journal.pone.0019969)
Supplement: Table S2 — The new thrombin inhibitors – derivatives of orcinol, their structures, SOL scoring function values and results of experimental testing. (DOC) [file pone.0019969.s002.doc]

**Table S2.** The new thrombin inhibitors – derivatives of orcinol, their structures, SOL scoring function values and results of experimental testing.

| **Ligand Type^1)^** | **N** | | **Structure** | **SOL scoring function, kcal/mol** | **Inhibition of thrombin activity (in buffer system)^2)^** | | **IC_50_ for ETP reduction in plasma, µM** | **IC_50(ETP)_/K_i_** |
| --- | --- | --- | --- | --- | --- | --- | --- | --- |
|  |  |  |  |  | **IC_50_, nM** | **K_i_^3)^, nM** |  |  |
| **The linker chain length – 3 carbon atoms (n = 3)** | | | | | | | | |
| **4-AP** | **4a** | |  | -6.42 | 10000 | 862 | 230 | 266.8 |
|  | **4b** | |  | -6.83 | 62000 | 5345 | 160 | 29.9 |
|  | **4c** | |  | -5.85 | 917 | 79.1 | 27 | 341.3 |
|  | **4d** | |  | -6.61 | 3400 | 293 | 40 | 136.5 |
| **2-AT** | **7a** | |  | -5.94 | 1800 | 155 | 25.8 | 166.5 |
|  | **7b** | |  | -6.07 | 800 | 69 | 8.6 | 124.6 |
|  | **7c** | |  | -5.54 | 300 | 25.9 | 9.3 | 359.1 |
| **IT** | **8a** | |  | -5.89 | 97 | 8.4 | 8 | 952.4 |
|  | **8e** | |  | -5.63 | 11.6 | 1.0 | 4.1 | 4100 |
|  | **8f** | |  | -5.81 | 23.5 | 2.03 | 1.24 | 610.8 |
|  | **8g** | |  | -5.42 | 5500 | 474 | 25.2 | 53.2 |
| **The linker chain length – 2 carbon atoms (n = 2)** | | | | | | | | |
| **4-AP** | **4e** |  | | -6.49 | 3.5 | 0.3 | 0.1 | 333.3 |
|  | **4f** |  | | -6.81 | 2.44 | 0.21 | 0.26 | 1238.1 |
|  | **4g** |  | | -6.60 | 8.7 | 0.75 | 0.16 | 213.3 |
|  | **4h** |  | | -7.03 | 9 | 0.78 | 0.15 | 192.3 |
|  | **4i** |  | | -6.56 | 9 | 0.78 | 0.25 | 320.5 |
|  | **4j** |  | | -6.75 | 11.6 | 1.0 | 4 | 4000 |
|  | **5^4)^** |  | | -5.45 | 589 | 50.8 | 2.5 | 49.2 |
|  | **6** |  | | -7.01 | 400  (S_FLUO_) | 46 | 1.6 | 34.8 |
| **2-AT** | **7d** |  | | -5.88 | 756 | 65.2 | 16 | 245.4 |
| **IT** | **8b** |  | | -5.48 | 17 | 1.5 | 0.8 | 533.3 |
|  | **8c** |  | | -6.02 | 3.8 | 0.33 | 0.16 | 484.8 |
|  | **8d** |  | | -6.12 | 11 | 0.95 | 0.9 | 947.4 |
|  | **8h** |  | | -5.61 | 65.8 | 5.7 | 0.53 | 93.0 |

^1)^ 4-Aminopyridinium (4-AP), isothiuronium (IT), and 2-aminothiazolinium (2-AT) derivatives.

^2)^ Activity was measured by hydrolysis of specific thrombin substrates in buffer system. The fast fluorogenic substrate BOC-Ala-Pro-Arg-AMC (S_FLUO_) was used only for investigation of compound **6**. In all other cases the chromogenic substrate (S_CHR_) was used.

^3)^ The K_I_ values were calculated with the assumption of a competitive type of inhibition.

^4)^ This substance may be assigned to 4-AP type only conventionally.
