# Supplementary material for: New Synthetic Thrombin Inhibitors: Molecular Design and Experimental Verification
Source: PLoS One. 2011 May 16;6(5):e19969. doi: 10.1371/journal.pone.0019969 (PMC3095642; doi:10.1371/journal.pone.0019969)
Supplement: Text S1 — Supplementary Materials and Methods. (DOC) [file pone.0019969.s003.doc]

**New Synthetic Thrombin Inhibitors: Molecular Design and Experimental Verification**

Elena I. Sinauridze, Alexey N. Romanov, Irina V. Gribkova, Olga A. Kondakova,

Stepan S. Surov, Aleksander S. Gorbatenko, Andrey A. Butylin, Mikhail Yu. Monakov,

Alexey A. Bogolyubov, Yuryi V. Kuznetsov, Vladimir B. Sulimov, Fazoyl I. Ataullakhanov

**Syntheses of the new thrombin inhibitors**

**Materials and Methods.**

All chemicals were purchased from Sigma-Aldrich Chemical Co or Acros Organics and were at least reagent grade. Compound **6** was synthesized previously **^(*)^**. Pyridine and acetonitrile were distilled twice over P_2_O_5_; acetone was dried over P_2_O_5_ for 20 minutes, decanted and distilled; diethyl ether was distilled once over KOH; triethylamine, tetrahydrofuran (THF) and dioxane were distilled over sodium wire; hexane, ethanol, chloroform, ethyl acetate, methyl tert-butyl ether (MTBE) and benzene were used without purification. Every time a drying procedure in vacuum (2 mm Hg of residual pressure) was used, the temperature at which the drying was carried out was 70-90^0^C and the time of the procedure was 2.5-3 hours unless otherwise specified, agents to absorb evacuated vapors in a vacuum trap were P_2_O_5_, KOH and paraffin according to the nature of the vapors. Thin-layer chromatography (TLC) was carried out with Merck 60F_254_ (0.25 mm thick) and Becker Silufol UV_254_ plates. Melting points were measured on Fisher Johns melting point apparatus and were not corrected. Elemental analysis was performed with combustion method in Laboratory of Microanalisys of Zelinsky Institute of Organic Chemistry RAS. ^1^H NMR spectra of final products were recorded on Brucker DRX500 (500 MHz) in DMSO-*d_6_* and for intermediates with Brucker AC200 (200 MHz) in CDCl_3_, chemical shifts were given in ppm relative to internal tetramethylsilane standard (TMS). ^1^H NMR spectra of iodoalkanes **3a-j** were not informative: signals of CH_2_I and CH_2_Cl protons overlapped particularly and other signals overlapped completely or particularly in all solvents we used. Mass spectra data were obtained with a MS-30 (Kratos), 70 eV. Substances **13a-j** did not give M^+^ in mass spectra: iodoalkanes are often unstable under electron impact; Ar–OCH_2_CH_2_–Br appeared to be not stable enough too. Liquid chromatography-mass spectrometry (LC-MS) data were obtained with Waters Aquiety System equipped PDA-detector and electrospray mass-spectrometer.

**3-(3-Chloropropoxy)-5-methylphenol** **(1a).** *Method A.* In 250 ml flask equipped with vacuum trap charged with P_2_O_5_, orcinol hydrate (3.00 g, 21.13 mmol) was heated at 60-80^0^C in vacuum for 1.5 h. (Orcinol was partially sublimated). Anhydrous potassium carbonate (6.10 g, 44.37 mmol), 1-bromo-3-chloropropane (2.1 ml, 3.33 g, 21.13 mmol) and acetone (60 ml) were added therein, and a reaction mixture was refluxed with stirring for 43 h, a solvent was evaporated, a residue was dissolved in water (100 ml), acidified with acetic acid to pH 7.5-8 and extracted with chloroform (3 × 15 ml). A chloroform extract was filtered, evaporated, and dried with sodium sulfate; a residue was chromatografically separated with benzene:ethyl acetate system of increasing polarity (from 1:0 to 19:1) on silica gel (5-40 µm) flash chromatography column (R_f_ 0.67, benzene/ethyl acetate 9/1), an oil obtained was dried in vacuum. Yield: 1.13 g (0.97 g of pure product, 25% of theoretical amount; a colorless oil residue is of 90% mol purity, major impurity according to ^1^H NMR is *m,m’*-(Cl–Alk–O)_2_C_6_H_3_–CH_3_). The compound was further used without purification. ^1^H NMR (CDCl_3_) 2.15-2.35 (m, 5H), 3.24 (t, 2H, *J* = 7.88 Hz), 4.09 (t, 2H, *J* = 7.50 Hz), 5.15 (br, 1H), 6.25 (s, 1H), 6.30 (s, 1H), 6.35 (s, 1H). Mass spectrum: 200 (M^+^), 201 (M^+^, ^35^Cl).

*Method B.* A mixture of orcinol hydrate (3.80 g, 27.00 mmol), 1-bromo-3-chloropropane (4.80 g, 30 mmol) and potassium carbonate (4.00 g, 29.00 mmol) was refluxed in acetonitrile (30 ml) with stirring for 36 h. A reaction mixture was evaporated, dissolved in ether (30 ml), washed with saturated solution of potassium carbonate (2 × 15 ml), water layer was discarded, and ether layer was extracted with solution of sodium hydroxide (10%, 3 × 15 ml). Ether layer was discarded, water layer was carefully acidified with concentrated hydrochloric acid to pH 3-4 and then extracted with ether (3 × 15 ml), ether extract was washed with small quantities of saturated solution of sodium hydrocarbonate (1-3%, 10 ml), with water (3 × 15 ml), dried with anhydrous sodium sulfate, diluted with approximately 1/3^rd^ part (by volume) with hexane and filtered through a layer of silica gel, a solvent was evaporated and a residue was dried in vacuum. Yield: 1.70 g (0.67 g of pure **1a**, 13% of theoretical amount; yellow oil, a mixture of about 70% mol orcinol and 30% mol of the product) **^(†)^**.

**3-(2-Chloroethoxy)-5-methylphenol** **(1b).** By analogy, according to *Method B* for **1a**, this compound was obtained from orcinol hydrate (100 mmol), 1-bromo-2-chloroethane (100 mmol) and potassium carbonate (120 mmol) after 32 h of reflux as yellow oil. Yield: 9.30 g (4.50 g of pure **1b**, 24% of theoretical amount, a mixture of about 50% mol of orcinol and 50% mol of the product). The compound was further used without purification. ^1^H NMR (CDCl_3_) 2.26 (s, 3H), 3.76 (t, 2H, *J* = 7.35 Hz), 4.16 (t, 2H, *J* = 7.35 Hz), 5.71 (br, 1H), 6.26 (s, 1H), 6.32 (s, 1H), 6.37 (s, 1H). Mass spectrum: 186 (M^+^), 187 (M^+^, ^35^Cl).

**3-(3-Chloropropoxy)-5-methylphenyl ester of benzenesulfonic acid** **(2a).** Benzene sulfochloride (3.00 g, 17.00 mmol) in THF (10 ml) was added to a solution of triethylamine (2.8 ml, 2.00 g, 20.00 mmol) and **1a** prepared by *Method B* (3.19 g containing 14.67 mmol of **1a**) in THF (20 ml), a mixture was stirred for 7 h at ambient temperature, quenched with water (0.1 ml) with stirring for 1 h at ambient temperature, a solution was evaporated; an oil was mixed with water (20 ml) and extracted with ether (3 × 15 ml), an ether solution was washed with aqueous sodium hydroxide (10%, 4 × 20 ml), with hydrochloric acid (3-5%, 20 ml) and with water (3 × 20 ml). Drying with anhydrous sodium sulfate and evaporation gave a yellow oil of 85% mol purity, major impurity according to ^1^H NMR is *m,m’*-(PhSO_2_O)_2_C_6_H_3_–CH_3_. Yield: 1.94 g (1.58 g of pure **2a**, 33% of theoretical amount) **^(‡)^**. ^1^H NMR (CDCl_3_) 2.10-2.26 (m, 5H), 3.70 (t, 2H, *J* = 6.47 Hz), 3.95 (t, 2H, *J_2_* = 5.39 Hz), 6.33 (s, 1H), 6.45 (s, 1H), 6.61 (s, 1H), 7.55 (t, 2H, *J*_3,2_ = 6.47 Hz, *J*_3,4_ = 8.62 Hz), 7.68 (t, 1H, *J*_4,3_ = 8.62 Hz, *J*_4,5_ = 8.62 Hz), 7.87 (d, 2H, *J*_2,3_ = 6.47 Hz). Mass spectrum: 340 (M^+^), 341 (M^+^, ^35^Cl).

**3-(3-Chloropropoxy)-5-methylphenyl ester of *p*-methylbenzenesulfonic acid** **(2b).** Tosylchloride (1.00 g, 5.24 mmol) was added to a solution of **1a** prepared by *Method A* (0.91 g containing 4.52 mmol of **1a**) in pyridine (15 ml) at 10-15^0^C, a mixture was stirred for 3 days at ambient temperature and then 2 h at 60^0^C; after cooling, water (1 ml) was added therein and a mixture was stirred for 0.5 h, the resulting mixture was poured into hydrochloric acid (5%, 100 ml), extracted with chloroform (3 × 15 ml), a chloroform solution was subsequently washed with hydrochloric acid (5%, 100 ml), water (15 ml), saturated sodium hydrocarbonate aqueous solution (15 ml) and water (15 ml), dried with sodium sulfate, evaporated and dried in vacuum. Yield: 1.66 g (1.53 g of pure **2b**, 95% of theoretical amount; colorless oil, 90% mol purity) **^(†)^**. ^1^H NMR (DMSO-*d*_6_) 2.09-2.23 (m, 5H), 2.42 (s, 3H,), 3.73 (t, 2H *J* = 6.80 Hz), 3.96 (t, 2H *J* = 5.49 Hz), 6.32 (s, 1H), 6.50 (s, 1H), 6.74 (s, 1H), 7.39 (d, 2H *J* = 6.60 Hz), 7.76 (d, 2H *J* = 6.60 Hz). Mass spectrum: 354 (M^+^), 355 (M^+^, ^35^Cl).

**3-(3-Chloropropoxy)-5-methylphenyl ester of *o*-fluorobenzenesulfonic acid** **(2c).** This compound was synthesized from **1a** prepared by *Method B* by approach used to obtain **2a**. Yield: 97% of theoretical amount; the crude product contained 65% of **2c ^(†)^**. ^1^H NMR (CDCl_3_) 2.01-2.29 (m, 5H ), 3.70 (t, 2H *J* = 7.11 Hz), 3.92 (t, 2H *J* = 5.89 Hz), 6.39 (s, 1H), 6.59 (s, 1H), 6.78 (s, 1H), 7.49 (t, 1H *J*_4,3_ = 7.90 Hz, *J*_4,5_ = 9.11 Hz), 7.68 (t, 1H *J*_5,4_ = 9.11 Hz, *J*_5,6_ = 7.90 Hz), 7.91 (dd, 1H *J*_3,4_ = 7.90 Hz, *J*_3,2_ = 1.83 Hz), 7.95 (dd, 1Н *J*_6,5_ = 7.90 Hz, *J*_6,2_ = 1.83 Hz). Mass spectrum: 358 (M^+^), 359 (M^+^, ^35^Cl).

**3-(3-Chloropropoxy)-5-methylphenyl ester of *o*-chlorobenzenesulfonic acid** **(2d).** **2d** was synthesized from **1a** prepared by *Method B* by approach used to obtain **2a**. Yield: 77% of theoretical amount. The crude product contained 65% of **2d ^(†)^**. ^1^H NMR (CDCl_3_) 2.01-2.29 (m, 5H ), 3.71 (t, 2H *J* = 7.08 Hz), 3.99 (t, 2H *J* = 6.12 Hz), 6.33 (s, 1H), 6.45 (s, 1H), 6.61 (s, 1H), 7.58 (t, 1H *J*_4,5_ = 7.93 Hz, *J*_4,3_ = 7.99 Hz), 7.84 (t, 1H *J*_5,4_ = 7.93 Hz, *J*_5,6_ = 7.92 Hz), 7.88 (d, 1H *J*_3,4_ = 7.99 Hz), 7.94 (d, 1Н *J*_6,5_ = 7.92 Hz). Mass spectrum: 374 (M^+^), 375 (M^+^, Cl^35^), 376 (M^+^, two ^35^Cl).

**3-(2-Chloroethoxy)-5-methylphenyl ester of *o*-fluorobenzenesulfonic acid** **(2e).** **2e** was synthesized from **1b** by approach used to obtain **2a**. Yield: 34% of theoretical amount **^(‡)^**. ^1^H NMR (CDCl_3_) 2.18 (s, 3H ), 3.79 (t, 2H *J* = 8.75 Hz), 4.20 (t, 2H *J* = 8.75 Hz), 6.39 (s, 1H), 6.49 (s, 1H), 6.69 (s, 1H), 7.49 (t, 1H *J*_4,3_ = 7.90 Hz, *J*_4,5_ = 9.11 Hz), 7.69 (t, 1H *J*_5,4_ = 9.11 Hz, *J*_5,6_ = 7.90 Hz), 7.92 (dd, 1H *J*_3,4_ = 7.90 Hz, *J*_3,2_ = 1.83 Hz), 7.99 (dd, 1Н *J*_6,5_ = 7.90 Hz, *J*_6,2_ = 1.83 Hz). Mass spectrum: 344 (M^+^), 345 (M^+^, ^35^Cl).

**3-(2-Chloroethoxy)-5-methylphenyl ester of *o*-chlorobenzenesulfonic acid** **(2f).** **2f** was synthesized from **1b** by approach used to obtain **2a**. Yield: 35% of theoretical amount **^(‡)^**. ^1^H NMR (CDCl_3_) 2.18 (s, 3H ), 3.75 (t, 2H *J* = 8.73 Hz), 4.18 (t, 2H *J* = 8.73 Hz), 6.32 (s, 1H), 6.42 (s, 1H), 6.63 (s, 1H), 7.59 (t, 1H *J*_4,5_ = 7.93 Hz, *J*_4,3_ = 7.99 Hz), 7.85 (t, 1H *J*_5,4_ = 7.93 Hz, *J*_5,6_ = 7.92 Hz), 7.89 (d, 1H *J*_3,4_ = 7.99 Hz), 7.99 (d, 1Н *J*_6,5_ = 7.92 Hz). Mass spectrum: 360 (M^+^), 361 (M^+^, ^35^Cl), 362 (M^+^, two ^35^Cl).

**3-(2-Chloroethoxy)-5-methylphenyl ester of *o*-(methylsulfonyl)benzenesulfonic acid** **(2g).** **2g** was synthesized from **1b** by approach used to obtain **2a**. Yield: 44% of theoretical amount **^(‡)^**. ^1^H NMR (CDCl_3_) 2.18 (s, 3H ), 3.76 (t, 2H *J* = 8.77 Hz), 4.19 (t, 2H *J* = 8.77 Hz), 6.37 (s, 1H), 6.46 (s, 1H), 6.69 (s, 1H), 7.80 (t, 1H *J* = 7.91), 8.01-8.10 (m, 2H ), 8.38 (d, 1H *J* = 8.50 Hz). Mass spectrum: 404 (M^+^), 405 (M^+^, ^35^Cl).

**3-(2-Chloroethoxy)-5-methylphenyl ester of *p*-chlorobenzenesulfonic acid (2h).** **2h** was synthesized from **1b** by approach used to obtain **2a**. Yield: 45% of theoretical amount **^(‡)^**. ^1^H NMR (CDCl_3_) 2.18 (s, 3H ), 3.78 (t, 2H *J* = 8.71 Hz), 4.21 (t, 2H *J* = 8.71 Hz), 6.39 (s, 1H), 6.49 (s, 1H), 6.69 (s, 1H), 7.65 (d, 2H *J* = 7.91 Hz), 7.89 (d, 2H *J* = 7.91 Hz). Mass spectrum: 360 (M^+^), 361 (M^+^, ^35^Cl), 362 (M^+^, two ^35^Cl).

**3-(2-Chloroethoxy)-5-methylphenyl ester of benzenesulfonic acid (2i).** **2i** was synthesized from **1b** by approach used to obtain **2a**. Yield: 72% of theoretical amount **^(‡)^**. ^1^H NMR (CDCl_3_) 2.18 (s, 3H ), 3.77 (t, 2H *J* = 8.71 Hz), 4.23 (t, 2H *J* = 8.71 Hz), 6.36 (s, 1H), 6.51 (s, 1H), 6.69 (s, 1H), 7.67 (t, 2H *J*_3,2_ = 6.51 Hz, *J*_3,4_ = 8.69 Hz), 7.81 (t, 1H *J*_4,3_ = 8.69 Hz, *J*_4,5_ = 8.69 Hz), 7.88 (d, 2H *J*_2,3_ = 6.51 Hz). Mass spectrum: 326 (M^+^), 327 (M^+^, ^35^Cl).

**3-(2-Chloroethoxy)-5-methylphenyl ester of *p*-methylbenzenesulfonic acid (2j).** **2j** was synthesized from **1b** by approach used to obtain **2a**. Yield: 46% of theoretical amount **^(‡)^**. ^1^H NMR (CDCl_3_) 2.18 (s, 3H ), 2.43 (s, 3H,), 3.75 (t, 2H *J* = 8.71 Hz), 4.18 (t, 2H *J* = 8.71 Hz), 6.38 (s, 1H), 6.49 (s, 1H), 6.68 (s, 1H), 7.39 (d, 2H *J* = 6.61 Hz), 7.75 (d, 2H *J* = 6.61 Hz). Mass spectrum: 340 (M^+^), 341 (M^+^, ^35^Cl).

**Iodoalkanes 3a-j: common protocol for chlorine to iodine exchange in 2a-j.** A crude chloroalkane **2a-j** (5.00 mmol) was refluxed with 13.00 mmol of dry sodium iodide in acetone (30 ml) for 48 h, a reaction mixture was diluted with hexane (30 ml), inorganic salts were filtered off and washed with hexane (3 × 15 ml), mother liquor and combined hexane washings were evaporated together and an oil obtained was dried in vacuum to give corresponding iodoalkane in 95-98% yield on pure starting chloroalkane – and therefore the molar ratio iodoalkane/*m*,*m’*-(ArSO_2_O)_2_C_6_H_3_–CH_3_ remains the same as in the initial starting chloroalkane. It slightly varies from experiment to experiment, so the ratio assumed to be 48:52 in all cases, excepting **2c** and **2d** where it is assumed to be 62.4:37.6. The assumption about complete conversion of chloroalkane was based on the weight of the separated inorganic salts. All the iodoalkanes were used in subsequent reactions without purification.

**Common protocol for preparation of 4-amino-1-substituted pyridinium salts 4a-j.** A crude iodoalkane **3a-j** (1.50 mmol) and 4-aminopyridine (0.145 g, 1.543 mmol) were refluxed for 20 h in 15 ml dioxane, and solvent was evaporated. A residue after evaporation was triturated with ether and the solidified product was twice crystallized from a mixture of dioxane and acetonitrile (5:1). The salts were dried in vacuum. Yields are given in percent to initial iodoalkane.

**4-Amino-1-{3-[3-methyl-5-(benzenesulfonyloxy)phenoxy]propyl-1}pyridinium iodide (4a). 4a** was prepared from **3a**. Yield: 78.0%. Mp 190-191^0^C. ^1^H NMR (DMSO-*d*_6_) 2.11-2.24 (m, 5H ), 3.88 (t, 2H *J* = 5.49 Hz), 4.25 (t, 2H *J* = 6.71 Hz), 6.31 (s, 1H), 6.44 (s, 1H), 6.66 (s, 1H), 6.82 (d, 2H *J* = 7.33 Hz), 7.68 (t, 2H *J*_3,2_ = 7.32 Hz, *J*_3,4_ = 7.91 Hz), 7.82 (t, 1H *J*_4,3_ = 7.91 Hz, *J_4,5_* = 7.91 Hz), 7.87 (d, 2H *J_2,3_* = 7.32 Hz), 8.09 (br, 2H ), 8.18 (d, 2H *J* = 7.33 Hz). Mass spectrum: 412 (M^+^).

**4-Amino-1-{3-[3-methyl-5-(4-methylbenzenesulfonyloxy)phenoxy]propyl-1}pyridinium iodide (4b).** This compound was prepared from **3b**. Yield: 85.1%. Mp 162-163^0^C. ^1^H NMR (DMSO-*d*_6_) 2.01-2.25 (m, 5H ), 2.43 (s, 3H,), 3.88 (t, 2H *J* = 5.49 Hz), 4.26 (t, 2H *J* = 6.71 Hz), 6.32 (s, 1H), 6.46 (s, 1H), 6.66 (s, 1H), 6.85 (d, 2H *J* = 5.90 Hz), 7.49 (d, 2H *J* = 6.51 Hz), 7.76 (d, 2H *J* = 6.51 Hz), 8.20 (d, 2H *J* = 5.90 Hz), 8.25 (br, 2H ). Mass spectrum: 426 (M^+^).

**4-Amino-1-{3-[3-methyl-5-(2-fluorobenzenesulfonyloxy)phenoxy]propyl-1}pyridinium iodide (4c). 4c** was prepared from **3c**. Yield: 49.6%. Mp 186-187^0^C. ^1^H NMR (DMSO-*d*_6_) 2.10-2.25 (m, 5H ), 3.91 (t, 2H *J* = 5.49 Hz), 4.26 (t, 2H *J* = 6.71 Hz), 6.39 (s, 1H), 6.50 (s, 1H), 6.69 (s, 1H), 6.80 (d, 2H *J* = 7.33 Hz), 7.46 (t, 1H *J*_4,3_ = 7.93 Hz, *J*_4,5_ = 9.15 Hz), 7.64 (t, 1H *J*_5,4_ = 9.15 Hz, *J*_5,6_ = 7.93 Hz), 7.81 (dd, 1H *J*_3,4_ = 7.93 Hz, *J*_3,2_ = 1.83 Hz), 7.91 (dd, 1Н *J*_6,5_ = 7.93 Hz, *J*_6,2_ = 1.83 Hz), 8.08 (br, 2H ), 8.17 (d, 2H *J* = 7.33 Hz). Mass spectrum: 416 (M^+^).

**4-Amino-1-{3-[3-methyl-5-(2-chlorobenzenesulfonyloxy)phenoxy]propyl-1}pyridinium iodide (4d). 4d** was prepared from **3d**. Yield: 65.1%. Mp 165-166^0^C. ^1^H NMR (DMSO-*d*_6_) 2.11-2.29 (m, 5H ), 3.91 (t, 2H *J* = 5.49 Hz), 4.26 (t, 2H *J* = 6.71 Hz), 6.40 (s, 1H), 6.50 (s, 1H), 6.68 (s, 1H), 6.80 (d, 2H *J* = 6.72 Hz), 7.59 (t, 1H *J*_4,3_ = 7.93 Hz, *J*_4,5_ = 7.94 Hz), 7.83 (t, 1H *J*_5,4_ = 7.94 Hz, *J*_5,6_ = 7.93 Hz), 7.87 (d, 1H *J*_3,4_ = 7.93 Hz), 7.95 (d, 1Н *J*_6,5_ = 7.93 Hz), 8.07 (br, 2H ), 8.17 (d, 2H *J* = 6.72 Hz). Mass spectrum: 432 (M^+^), 433 (M^+^, ^35^Cl).

**4-Amino-1-{2-[3-methyl-5-(2-fluorobenzenesulfonyloxy)phenoxy]ethyl-1}pyridinium iodide (4e). 4e** was prepared from **3e**. Yield: 36.2%. Mp 198-199^0^C. ^1^H NMR (DMSO-*d*_6_) 2.20 (s, 3H), 4.26 (t, 2H *J* = 4.28 Hz), 4.49 (t, 2H *J* = 4.28 Hz), 6.46 (s, 1H), 6.51 (s, 1H), 6.75 (s, 1H), 6.83 (d, 2H *J* = 6.42 Hz), 7.44 (t, 1H *J*_4,3_ = 7.93 Hz, *J*_4,5_ = 9.15 Hz), 7.63 (t, 1H *J*_5,4_ = 9.15 Hz, *J*_5,6_ = 7.93 Hz), 7.80 (dd, 1H *J_3,4_* = 7.93 Hz, *J*_3,2_: see note **^(#)^**) 7.92 (dd, 1Н *J_6,5_* = 7.32 Hz, *J*_6,2_: see note **^(#)^**), 8.18 (br, 2H ), 8.18 (d, 2H *J* = 6.42 Hz). Mass spectrum: 402 (M^+^). Calculated, % C (45.29), H (3.80); Found, % C (45.37), H (3.96).

**4-Amino-1-{2-[3-methyl-5-(2-chlorobenzenesulfonyloxy)phenoxy]ethyl-1}pyridinium iodide (4f). 4f** was prepared from **3f**. Yield: 58.1%. Mp 130-137^0^C. ^1^H NMR (DMSO-*d*_6_) 2.20 (s, 3H ), 4.26 (t, 2H *J* = 4.88 Hz), 4.49 (t, 2H *J* = 4.88 Hz), 6.45 (s, 1H), 6.51 (s, 1H), 6.74 (s, 1H), 6.82 (d, 2H *J* = 7.33 Hz), 7.58 (t, 1H *J*_4,5_ = 7.93 Hz, *J*_4,3_ = 7.94 Hz), 7.84 (t, 1H *J*_5,4_ = 7.93 Hz, *J*_5,6_ = 7.94 Hz), 7.88 (d, 1H *J_3,4_* = 7.94 Hz), 7.94 (d, 1Н *J_6,5_* = 7.94 Hz), 8.14 (br, 2H ), 8.18 (d, 2H *J* = 7.33 Hz). Mass spectrum: 418 (M^+^), 419 (M^+^, ^35^Cl). Calculated, % C (43.93), H (3.69); Found, % C (44.45), H (3.81).

**4-Amino-1-{2-[3-methyl-5-(2-(methylsulfonyl)benzenesulfonyloxy)phenoxy]ethyl-1}pyridinium iodide (4g).** This compound was prepared from **3g**. Yield: 45.2%. Mp 120-121^0^C. ^1^H NMR (DMSO-*d*_6_) 2.21 (s, 3H), 3.47 (s, 3H), 4.26 (t, 2H *J* = 4.88 Hz), 4.49 (t, 2H *J* = 4.88 Hz), 6.51 (s, 1H), 6.57 (s, 1H), 6.75 (s, 1H), 6.82 (d, 2H *J* = 7.02 Hz), 7.95 (t, 1H *J* = 7.93), 8.10-8.13 (m, 4H), 8.18 (d, 2H *J* = 7.02 Hz), 8.38 (d, 1H *J* = 8.55 Hz). Mass spectrum: 462 (M^+^). Calculated, % C (42.72), H (3.93); Found, % C (43.29), H (4.16).

**4-Amino-1-{2-[3-methyl-5-(4-chlorobenzenesulfonyloxy)phenoxy]ethyl-1}pyridinium iodide (4h). 4h** was prepared from **3h**. Yield: 15.2%. Mp 127-128^0^C. ^1^H NMR (DMSO-*d*_6_) 2.21 (s, 3H ), 4.26 (t, 2H *J* = 4.27 Hz), 4.49 (t, 2H *J* = 4.27 Hz), 6.47 (s, 1H), 6.49 (s, 1H), 6.75 (s, 1H), 6.83 (d, 2H *J* = 6.72 Hz), 7.76 (d, 2H *J* = 7.93 Hz), 7.89 (d, 2H *J* = 7.93 Hz), 8.15-8.25 (m, 4H). Mass spectrum: 418 (M^+^), 419 (M^+^, ^35^Cl). Calculated, % C (43.93), H (3.69); Found, % C (44.37), H (3.72).

**4-Amino-1-{2-[3-methyl-5-(benzenesulfonyloxy)phenoxy]ethyl-1}pyridinium iodide (4i).** This compound was prepared from **3i**. Yield: 60.1%. Mp 195-196^0^C. ^1^H NMR (DMSO-*d*_6_) 2.20 (s, 3H ), 4.24 (t, 2H *J* = 4.88 Hz), 4.48 (t, 2H *J* = 4.88 Hz), 6.39 (s, 1H), 6.45 (s, 1H), 6.73 (s, 1H), 6.82 (d, 2H *J* = 7.33 Hz), 7.68 (t, 2H *J*_3,2_ = 7.32 Hz, *J*_3,4_ = 7.93 Hz), 7.82 (t, 1H *J*_4,3_ = 7.93 Hz, *J*_4,5_ = 7.93 Hz), 7.87 (d, 2H *J*_2,3_ = 7.32 Hz), 8.14 (br, 2H ), 8.18 (d, 2H *J* = 7.33 Hz). Mass spectrum: 384 (M^+^). Calculated, % C (46.88), H (4.13); Found, % C (47.04), H (4.27).

**4-Amino-1-{2-[3-methyl-5-(4-methylbenzenesulfonyloxy)phenoxy]ethyl-1}pyridinium iodide (4j). 4j** was prepared from **3j**. Yield: 32.2%. Mp 125-126^0^C. ^1^H NMR (DMSO-*d*_6_) 2.20 (s, 3H ), 2.43 (s, 3H,), 4.26 (t, 2H *J* = 4.27 Hz), 4.49 (t, 2H *J* = 4.27 Hz), 6.43 (s, 1H), 6.45 (s, 1H), 6.72 (s, 1H), 6.83 (d, 2H *J* = 7.32 Hz), 7.48 (d, 2H *J* = 7.93 Hz), 7.75 (d, 2H *J* = 7.93 Hz), 8.19 (d, 2H *J* = 7.32 Hz), 8.18 (br, 2H ). Mass spectrum: 398 (M^+^).

**4-Amino-1-{2-[3-methyl-5-(benzenesulfonyloxy)phenoxy]ethyl-1}pirimidinium iodide (5).** Crude iodoalkane **3i** (0.80 g containing 0.88 mmol of pure **3i**) and 4-aminopirimidine (0.084 g, 0.88 mmol) were refluxed in dioxane (5 ml) for 14 h, dioxane was evaporated, a residue was washed with ether (4 × 5 ml) and dried in vacuum for 5 h at 90-100^0^C with paraffin trap. Yield: 17.7% (as percentage of 4-aminopirimidine). Mp 194-195^0^C. ^1^H NMR (DMSO-*d*_6_) 2.19 (s, 3H ), 4.26 (t, 2H *J* = 4.88 Hz), 4.46 (t, 2H *J* = 4.88 Hz), 6.43 (s, 1H), 6.45 (s, 1H), 6.74 (s, 1H), 6.78 (d, 1H , *J* = 7.32 Hz), 7.58 (t, 1H *J*_4,3_ = 7.94 Hz, *J_4,5_* = 7.94 Hz), 7.83 (t, 2H *J_3,2_* = 7.39 Hz, *J_3,4_* = 7.94 Hz), 7.87 (d, 1H *J_2,3_* = 7.39 Hz), 7.94 (d, 1H *J*_6,5_ = 7.39 Hz), 8.28 (d, 1H , *J* = 7.32 Hz), 8.79 (c, 1H ), 9.04 (s, 2H ). Mass spectrum: 385 (M^+^).

**Common protocol for preparation of 2-amino-3-substituted thiazolium salts 7a-d.** A сrude iodoalkane (0.52 mmol **3a,c,d, or f)** and 2-aminothiazole (0.040 g, 0.40 mmol) were refluxed in dioxane (10 ml) for 24 h, a solvent was evaporated. A residue after evaporation was triturated with ether and the solidified product was twice crystallized from a mixture of dioxane and acetonitrile (6:1), crystals were washed with ether (3 × 5 ml) and dried in vacuum. Yields are given as percentages of iodoalkane.

**2-Amino-1-{3-[3-methyl-5-(benzenesulfonyloxy)phenoxy]propyl-1}thiazolium iodide (7a). 7a** was prepared from **3a**. Yield: 65.1%. Mp 98-100^0^C. ^1^H NMR (DMSO-*d*_6_) 2.21 (s, 3H ), 2.13 (m, 2H ), 3.96 (t, 2H *J* = 5.55 Hz), 4.16 (t, 2H *J* = 6.02 Hz), 6.41 (s, 1H), 6.50 (s, 1H), 6.73 (s, 1H), 7.03 (d, 1H, *J* = 4.27 Hz), 7.41 (d, 1H, *J* = 4.27 Hz), 7.57 (t, 1H *J*_4,5_ = 7.94 Hz, *J*_4,3_ = 7.94 Hz), 7.80 (t, 2H *J*_3,4_ = 7.94 Hz, *J*_3,2_ = 6.89 Hz), 7.87 (d, 1H *J*_6,5_ = 6.89 Hz), 7.94 (d, 1H *J*_2,3_ = 6.89 Hz), 9.39 (s, 2H ). Mass spectrum: 390 (M^+^).

**2-Amino-1-{3-[3-methyl-5-(2-fluorobenzenesulfonyloxy)phenoxy]propyl-1}thiazolium iodide (7b). 7b** was prepared from **3c**. Yield: 30.0%. Mp 110-111^0^C. ^1^H NMR (DMSO-*d*_6_) 2.22 (s, 3H ), 2.12 (m, 2H ), 3.95 (t, 2H *J* = 6.10 Hz), 4.15 (t, 2H *J* = 6.71 Hz), 6.41 (s, 1H), 6.50 (s, 1H), 6.70 (s, 1H), 7.02 (d, 1H, *J* = 4.58 Hz), 7.42 (d, 1H, *J* = 4.58 Hz), 7.46 (t, 1H *J*_4,3_ = 7.93 Hz, *J*_4,5_ = 9.15 Hz), 7.64 (t, 1H *J*_4,5_ = 9.15 Hz, *J*_4,5_ = 7.93 Hz), 7.81 (dd, 1H *J*_3,4_ = 7.93 Hz, *J*_3,2_ = 1.83 Hz), 7.91 (dd, 1Н *J*_6,5_ = 7.93 Hz, *J*_6,2_ = 1.83 Hz), 9.39 (s, 2H ). Mass spectrum: 408 (M^+^).

**2-Amino-1-{3-[3-methyl-5-(2-chlorobenzenesulfonyloxy)phenoxy]propyl-1}thiazolium iodide (7c). 7c** was prepared from **3d**. Yield: 53.0%. Mp 160-162^0^C. ^1^H NMR (DMSO-*d*_6_) 2.21 (s, 3H ), 2.12 (m, 2H ), 3.95 (t, 2H *J* = 5.50 Hz), 4.15 (t, 2H *J* = 6.10 Hz), 6.42 (s, 1H), 6.51 (s, 1H), 6.70 (s, 1H), 7.01 (d, 1H, *J* = 4.27 Hz), 7.42 (d, 1H, *J* = 4.27 Hz), 7.59 (t, 1H *J*_4,5_ = 7.94 Hz, *J*_4,3_ = 7.93 Hz), 7.83 (t, 1H *J*_5,6_ = 7.93 Hz, *J*_5,4_ = 7.94 Hz), 7.88 (d, 1H *J*_3,4_ = 7.93 Hz), 7.95 (d, 1H *J*_6,5_ = 7.93 Hz), 9.39 (s, 2H ). Mass spectrum: 424 (M^+^), 425 (M^+^, ^35^Cl).

**2-Amino-1-{2-[3-methyl-5-(2-chlorobenzenesulfonyloxy)phenoxy]ethyl-1}thiazolium iodide (7d). 7d** was prepared from **3f**. Yield: 35.0%. Mp 150-151^0^C. ^1^H NMR (DMSO-*d*_6_) 2.21 (s, 3H ), 4.21 (t, 2H *J* = 4.88 Hz), 4.37 (t, 2H *J* = 4.88 Hz), 6.44 (s, 1H), 6.52 (s, 1H), 6.73 (s, 1H), 6.96 (d, 1H, *J* = 4.27 Hz), 7.38 (d, 1H, *J* = 4.27 Hz), 7.58 (t, 1H *J*_4,5_ = 7.94 Hz, *J*_4,3_ = 7.94 Hz), 7.83 (t, 1H *J*_5,6_ = 7.93 Hz, *J*_5,4_ = 7.94 Hz), 7.87 (d, 1H *J*_3,4_ = 7.94 Hz), 7.94 (d, 1Н *J*_6,5_ = 7.93 Hz), 9.39 (s, 2H ). Mass spectrum: 410 (M^+^), 411 (M^+^, ^35^Cl).

**Common protocol for preparation of isothiuronium salts 8a-g.** Isothiuronium salts were prepared by the method used to obtain thiazolium salts from thiocarbamide or methyl thiocarbamide and corresponding iodoalkanes (**3a,c,d,f,g, or i)** but in this case products were crystallized from dioxane. Purification of **8c,d** has some minor differences. Yields are given as percentage of iodoalkane.

**S-{3-[3-methyl-5-(benzenesulfonyloxy)phenoxy]propyl-1}isothiuronium iodide (8a). 8a** was prepared from **3a** and thiocarbamide. Yield: 80.1%. Mp 128-130^0^C. ^1^H NMR (DMSO-*d*_6_) 1.92-2.17 (m, 2H ), 2.22 (s, 3H ), 3.95 (t, 2H, *J* = 6.10 Hz), 4.26 (t, 2H *J* = 7.33 Hz), 6.47 (s, 1H), 6.50 (s, 1H), 6.75 (s, 1H), 7.57 (t, 1H *J*_4,3_ = 7.94 Hz, *J*_4,5_ = 7.94 Hz), 7.80 (t, 2H *J*_3,2_ = 6.94 Hz, *J*_3,4_ = 7.94 Hz), 7.88 (d, 1H *J*_6,5_ = 6.94 Hz), 7.94 (d, 1H *J*_2,3_ = 6.94 Hz), 9.03 (br s, 4H). Mass spectrum: 380 (M^+^).

**S-{2-[3-methyl-5-(2-(methylsulfonyl)benzenesulfonyloxy)phenoxy]ethyl-1}isothiuronium iodide (8b). 8b** was prepared from **3g** and thiocarbamide. Yield: 18.2%. Mp 90-92^0^C. ^1^H NMR (DMSO-*d*_6_) 2.22 (s, 3H ), 3.48 (s, 3H, SO_2_C*H*_3_), 3.55 (t, 2H, *J* = 6.11 Hz), 4.13 (t, 2H *J* = 6.11 Hz), 6.53 (s, 1H), 6.59 (s, 1H), 6.78 (s, 1H), 7.97 (t, 1H *J* = 7.94 Hz), 8.10-8.13 (m, 2H ), 8.38 (d, 1H *J* = 7.94 Hz), 9.07 (br s, 4H ). Mass spectrum: 444 (M^+^).

**S-{2-[3-methyl-5-(2-chlorobenzenesulfonyloxy)phenoxy]ethyl-1}isothiuronium iodide (8c).** This compound was prepared from **3f** and thiocarbamide. Purification: after evaporation of dioxane, a crude product was washed with cold water (1 ml per mmol), dried in vacuum at ambient temperature for 6 h with trap filled with P_2_O_5_, crystallized from dioxane, washed with ether (10 × 5 ml) and dried again according to the standard protocol. Yield: 69.0%. Mp 100-102^0^C. ^1^H NMR (DMSO-*d*_6_) 2.22 (s, 3H ), 3.54 (t, 2H, *J* = 5.52 Hz), 4.14 (t, 2H *J* = 5.52 Hz), 6.48 (s, 1H), 6.52 (s, 1H), 6.77 (s, 1H), 7.59 (t, 1H *J*_4,3_ = 7.94 Hz, *J*_4,5_ = 7.93 Hz), 7.83 (t, 1H *J*_5,6_ = 7.94 Hz, *J*_5,4_ = 7.93 Hz), 7.87 (d, 1H *J*_3,4_ = 7.94 Hz), 7.95 (d, 1H *J*_6,5_ = 7.94 Hz), 9.02 (br, 4H ). Mass spectrum: 414 (M^+^), 415 (M^+^, ^35^Cl). Calculated, % C (36.84), H (3.43); Found, % C (36.84), H (3.60).

**S-{2-[3-methyl-5-(benzenesulfonyloxy)phenoxy]ethyl-1}isothiuronium iodide (8d). 8d** was prepared from **3i** and thiocarbamide. Purification: the substance was crystallized from a mixture dioxane and acetonitrile (7:1) washed with ether (5 × 5 ml) and dried. Yield: 45.1%. Mp 130-132^0^C. ^1^H NMR (DMSO-*d*_6_) 2.22 (s, 3H ), 3.54 (t, 2H, *J* = 5.49 Hz), 4.11 (t, 2H *J* = 5.49 Hz), 6.41 (s, 1H), 6.48 (s, 1H), 6.76 (s, 1H), 7.69 (t, 2H *J*_3,2_ = 7.32 Hz, *J*_3,4_ = 7.95 Hz), 7.84 (t, 1H *J*_4,3_ = 7.95 Hz, *J*_4,5_ = 7.95 Hz), 7.89 (d, 2H *J*_2,3_ = 7.32 Hz), 9.01 (br, 4H ). Mass spectrum: 366 (M^+^).

**S-{3-[3-methyl-5-(2-chlorobenzenesulfonyloxy)phenoxy]propyl-1}isothiuronium iodide (8e). 8e** was prepared from **3d** and thiocarbamide. Yield: 55.0%. Mp 91-92^0^C. ^1^H NMR (DMSO-*d*_6_) 1.90-2.15 (m, 2H ), 2.22 (s, 3H ), 3.97 (t, 2H, *J* = 6.10 Hz), 4.26 (t, 2H *J* = 7.33 Hz), 6.47 (s, 1H), 6.51 (s, 1H), 6.75 (s, 1H), 7.60 (t, 1H *J*_4,3_ = 7.93 Hz, *J*_4,5_ = 7.94 Hz), 7.84 (t, 1H *J*_5,6_ = 7.94 Hz, *J*_5,4_ = 7.94 Hz), 7.88 (d, 1H *J*_3,4_ = 7.93 Hz), 7.96 (d, 1Н *J*_6,5_ = 7.94 Hz), 8.95, 9.07 (2br, 4H ). Mass spectrum: 414 (M^+^), 415 (M^+^, ^35^Cl).

**S-{3-[3-methyl-5-(2-fluorobenzenesulfonyloxy)phenoxy]propyl-1}isothiuronium iodide (8f). 8f** was prepared from **3c** and thiocarbamide. Yield: 57.2%. Oil or glass-like substance. ^1^H NMR (DMSO-*d*_6_) 1.90-2.15 (m, 2H ), 2.22 (s, 3H ), 3.25 (t, 2H, *J* = 6.71 Hz), 3.98 (t, 2H *J* = 6.10 Hz), 6.41 (s, 1H), 6.50 (s, 1H), 6.76 (s, 1H), 7.46 (t, 1H *J*_4,3_ = 7.33 Hz, *J*_4,5_ = 9.15 Hz), 7.64 (t, 1H *J*_5,4_ = 9.15 Hz, *J*_5,6_ = 7.32 Hz), 7.82 (dd, 1H *J*_3,4_ = 7.33, *J*_3,2_: see note ^(#)^), 7.92 (dd, 1Н *J*_6,5_ = 7.33, *J*_6,2_: see note ^(#)^), 9.04 (br s, 4H ). Mass spectrum: 398 (M^+^).

**S-{3-[3-methyl-5-(2-chlorobenzenesulfonyloxy)phenoxy]propyl-1}-N-methylisothiuronium iodide (8g).** This compound was prepared from **3d** and methythiocarbamide. Yield: 79.1%. Mp 85-87^0^C. ^1^H NMR (DMSO-*d*_6_) 1.95-2.06 (m, 2H ), 2.22 (s, 3H ), 2.91 (s, 3H, NHC*H*_3_), 3.23 (t, 2H, *J* = 6.72 Hz), 3.98 (t, 2H *J* = 6.12 Hz), 6.41 (s, 1H), 6.50 (s, 1H), 6.76 (s, 1H), 7.59 (t, 1H *J*_4,5_ = 7.93 Hz, *J*_4,3_ = 7.93 Hz), 7.84 (t, 1H *J*_5,6_ = 7.93 Hz, *J*_5,4_ = 7.94 Hz), 7.88 (d, 1H *J*_3,4_ = 7.93 Hz), 7.96 (d, 1Н *J*_6,5_ = 7.93 Hz), 9.00 (br s, 1H), 9.28(br, 1H), 9.54 (br, 1H). Mass spectrum: 428 (M^+^), 429 (M^+^, ^35^Cl).

**S-{2-[3-methyl-5-(2-chlorobenzenesulfonyloxy)phenoxy]ethyl-1}-N-methylisothiuronium iodide (8h). 8h** was prepared from **3f** and methylthiocarbamide. Yield: 34.5%. Mp 65-75^0^C. Hygroscopic substance. ^1^H NMR (DMSO-*d*_6_) 2.22 (s, 3H ), 2.91 (s, 3H, NHC*H*_3_), 3.61 (t, 2H, *J* = 5.49 Hz), 4.14 (t, 2H *J* = 5.49 Hz), 6.45 (s, 1H), 6.53 (s, 1H), 6.76 (s, 1H), 7.59 (t, 1H *J*_4,3_ = 7.94 Hz, *J*_4,5_ = 7.93 Hz), 7.84 (t, 1H *J*_5,6_ = 7.94 Hz, *J*_5,4_ = 7.93 Hz), 7.88 (d, 1H *J*_3,4_ = 7.94 Hz), 7.96 (d, 1H *J*_6,5_ = 7.94 Hz), 9.00 (br, 1H), 9.28 (br s, 1H), 9.543(br, 1H). Mass spectrum: 414 (M^+^), 415 (M^+^, ^35^Cl).

All newly synthesized thrombin inhibitors were examined experimentally. The obtained results are presented in Table S2.

.

**Notes**

**^(*)^ Synthesis had been described in:** Sinauridze EI, Ataullakhanov FI, Butylin AA, Sulimov VB, Romanov AN, et al. New compounds with antithrombin function and pharmaceutical compositions based on them. International Patent Application PCT/RU2008/000400, 27.06.2008 (priority 28.06.2007). Applicant: Bionika, Moscow, Russia.

**^(†)^** The compound was used in subsequent transformations without purification.

**^(‡)^** A mixture contains approximately equal molar quantities of 3-(ω-chloroalkoxy)-5-methylphenyl ester of arylsulfonic acid and diarylsulfonic ester of orcinol according to ^1^H NMR. Their ratio varies from 48:52 to 52:48 from substance to substance and from experiment to experiment, so the ratio assumed to be 1:1 in all cases unless otherwise is specified. The compound was the oil, and was used without purification.

**^(#)^** Signals look like broad triplet and quartet since they were unresolved due to the small constants.
